# The Senescence-Malignancy Axis: An Inflammaging Perspective

**DOI:** 10.3390/antiox15101357

**Published:** 2026-10-20

**Authors:** Irene Patrono, Margherita Sonnessa, Marco Trerotola, Anna Maria Giudetti, Simona De Summa, Francesco Alfredo Zito, Concetta Saponaro, Daniele Vergara

**Affiliations:** 1 Pathology Department, IRCCS Istituto Tumori “Giovanni Paolo II”, 70124 Bari, Italym.sonnessa@oncologico.bari.it (M.S.); a.zito@oncologico.bari.it (F.A.Z.); 2 Department of Medical, Oral and Biotechnological Sciences, University “G. D’Annunzio” of Chieti-Pescara, 66100 Chieti, Italy; marco.trerotola@unich.it; 3 Center for Advanced Science and Technologies (CAST), University “G. D’Annunzio” of Chieti-Pescara, 66100 Chieti, Italy; 4 Department of Biological and Environmental Sciences and Technologies (DiSTeBA), University of Salento, 73100 Lecce, Italy; anna.giudetti@unisalento.it (A.M.G.); daniele.vergara@unisalento.it (D.V.); 5 Molecular Diagnostics and Pharmacogenetics Unit, IRCCS Istituto Tumori “Giovanni Paolo II”, 70124 Bari, Italy; s.desumma@oncologico.bari.it

**Keywords:** aging, inflammaging, immune-senescence, geroncology

## Abstract

The transition from physiological cellular senescence to malignancy is not an isolated event, but a gradual, systemic remodeling of the biological landscape orchestrated by the persistent state of inflammaging. As detailed in this review, senescent populations do not remain passive. Through the chronic deployment of the senescence-associated secretory phenotype (SASP), they actively dismantle tissue architecture and convert healthy organs into a highly permissive pro-tumorigenic “soil”. This cross-talk is mediated by a complex network of signaling cascades including the IL-1 family, IL-6 and TNF-α, CXCL10 and CCL2-CCR2 chemokine axes, and MMPs. Crucially, this cellular decay is reinforced by a profound metabolic shift toward a hyper-glycolytic “metabolic SASP,” where extracellular lactate accumulation and prostaglandin synthesis permanently sustain the senescent state. Cellular senescence through the persistent accumulation of senescent cells and the accompanying age-related decline in immune surveillance drive “inflammaging”, a chronic, low-grade systemic inflammation that serves as a primary mechanistic bridge fueling the aging–cancer cycle.

## 1. Senescence and Inflammation Drive the Aging-Cancer Cycle

Aging is a non-linear biological process driven by the loss of homeostatic control, leading to a progressive accumulation of molecular and cellular damage. This process does not accelerate steadily; rather, it occurs in distinct waves during specific life stages and varies significantly across different organs and cells [1].

Although significant increases in longevity have occurred in recent decades, this has not been accompanied by equivalent progress in healthy longevity, giving rise to a gap between lifespan and healthy lifespan. While this physiological disparity remains a global phenomenon, its intensity and impact on clinical morbidity exhibit significant geographic heterogeneity, with current estimates suggesting a mean divergence of nearly a decade [2].

In this scenario, aging biomarkers have become essential tools to monitor age-related processes and highlight the disparity between an individual’s physiological condition (biological age) and the mere passage of time (chronological age) [3]. Building on the utility of these circulating biomarkers for real-time tracking, recent advancements have focused on their application within aging research, specifically to quantify the critical distinction between chronological and biological age. Biological age reflects the actual physiological state and functional decline of the body’s systems. Chronological age provides only a limited representation of the aging process, which is influenced by a multitude of genetic and environmental factors [4]. Biological age can be estimated through mathematical models that use biomarkers as input variables and chronological age as the reference outcome [5]. A compelling dimension of this research explores the intersection of premature physiological decline and the pathogenesis of age-related disorders. The most robust correlations have emerged within the field of neurodegeneration, where clinical research has introduced the “brain age gap” (BAG) as a metric for mapping systemic vulnerability [6]. In this model, a positive BAG correlates with an accelerated aging phenotype, while negative values indicate physiological resilience.

This approach has also been applied to cancer, although the mechanistic interaction between biological aging and neoplasia remains a more complex paradigm. The extent to which aging drives cancer development and the impact of cancer and its treatments on biological aging recently defined a dynamic model known as the “Aging–Cancer Cycle” [7]. According to this paradigm, physiological aging functions as a fundamental driver of oncogenesis, which subsequently triggers a feedback loop of biological acceleration that exacerbates clinical frailty and chronic morbidity.

When examining the convergence of phenotypic aging and cancer risk across broad populations, the PhenoAge algorithm emerges as a robust algorithm for quantifying biological age-related malignancy risk. Formulated by Levine and colleagues [8], PhenoAge has established that individuals exhibiting elevated biological age scores possess a significantly heightened susceptibility to malignant transformation within the UK Biobank consortium [9].

Further interrogation of over 150,000 participants from this cohort, categorized by generation-specific patterns, utilized the PhenoAge model to demonstrate that more contemporary birth groups experience accelerated aging. This biological acceleration functions as a primary driver for the rising incidence of early-onset malignancies (diagnosed before age 55), with a particular emphasis on pulmonary, digestive, and endometrial carcinomas. Moreover, data on organ-specific plasma proteomic aging patterns and their connections with the risk of early-onset cancers suggest that accelerated immune tissue aging is inextricably linked to early-onset pulmonary malignancy, whereas adipose tissue aging parallels early-onset colorectal oncogenesis. This result points to a potential functional link between organ aging and the risk of developing specific types of cancer, thereby drawing attention to systemic communication mechanisms that might be lost during the aging process [10].

Elucidating the specific mechanistic alterations that occur during organ-level aging is critical for understanding the progression toward oncogenic transformation. As demonstrated by Angarola and colleagues, the progressive dysregulation of the inflammatory landscape functions as a potent driver of tumor development and tissue remodeling. To investigate the functional effects of aging at the tissue level from a mechanistic perspective, the authors performed single-cell sequencing analyses on mammary glands from young adult and aged virgin female C57BL/6J mice. Data revealed that the aging process radically reconfigures mammary tissue, transforming it into a permissive, pro-tumorigenic niche. This organ-specific remodeling extends beyond the epithelial compartment, actively recruiting stromal populations and altering the immune microenvironment to promote neoplastic progression. The analysis highlighted variations in the cellular states of various cell types, revealing common age-related changes, including the dysregulation of cellular function and plasticity, and an upregulation of inflammation and senescence markers [11]. Elucidating these distinct mechanistic alterations provides critical insight into the progression toward malignant transformation, underscoring how progressive dysregulation of the inflammatory microenvironment functions as a potent driver of oncogenesis. A defining hallmark of this age-associated physiological decline is inflammaging, a smoldering, persistent systemic inflammatory state primarily orchestrated by senescence [12]. In oncology, the effects of senescent cells are profoundly context-dependent and double-edged, spanning tumor-suppressive growth arrest and tumor-promoting tissue remodeling [13]. While senescence serves as a vital safeguard by permanently halting the growth of pre-malignant cells [4], failure to clear senescent cells permits their persistence and can paradoxically promote disease progression. The senescence process affects not only tumor cells but the entire surrounding tumor microenvironment (TME). In various tumor contexts, senescent cells within the TME contribute to tumor progression and immunosuppression [14]. This pro-tumorigenic activity is largely driven by the senescence-associated secretory phenotype (SASP), through which senescent cells actively remodel the surrounding tissue into a permissive niche [15,16]. SASP composition varies according to the cell type, stimulus, tissue context, and duration of the senescent state. It comprises a mixture of bioactive cytokines, chemokines, growth regulators, matrix-remodeling proteases, and other proteins and metabolites [17,18]. Prominent SASP components include the pro-inflammatory interleukins (IL-6 and IL-8), the chemokine CCL2, and several matrix metalloproteinases (MMPs) that can sustain chronic inflammation and facilitate angiogenesis, tissue remodeling, and tumor-cell plasticity [17]. Importantly, cellular senescence is also a mechanism of resistance to chemotherapy. Many DNA-damaging chemotherapies induce senescence in both malignant cells and nonmalignant microenvironments. When therapy-induced senescent cells persist, they can constitute a viable, drug-tolerant reservoir and act through autocrine and paracrine SASP signaling to promote survival, cellular plasticity, stemness, epithelial–mesenchymal transition (EMT), and treatment resistance in neighboring tumor cells. Moreover, subsets of senescent tumor cells can escape cell-cycle arrest and resume proliferation with enhanced clonogenic and tumor-initiating capacity, whereas chemotherapy-induced senescence in stromal cells can generate a protective niche that supports residual disease, metastatic dissemination, and relapse. Recently, cisplatin-induced senescence in lung and ovarian cancer models was shown to generate a TGFβ-enriched SASP that activates TGFBR1–AKT/mTOR signaling and promotes resistance to platinum-based treatment [19].

This review explores the functional interplay connecting cellular senescence, chronic systemic inflammation, and cancer. Special emphasis is devoted to how reactive oxygen species (ROS) drive senescence and trigger the senescence-associated secretory phenotype (SASP). Furthermore, we evaluate the pro-tumorigenic secretome within the microenvironmental landscape, discussing novel translational avenues and targeted therapeutic prospects.

## 2. The Biological Axes of Aging: Cellular Senescence, Immunosenescence and Inflammaging

Cellular senescence is a state in which both immune and non-immune cells are characterized by permanent cell-cycle arrest in response to stress or damage [20]. Rather than a static state, cellular senescence is a highly dynamic, context-dependent trajectory, characterized by continuously evolving cell phenotypes and a permanent, mitogen-resistant cell-cycle arrest [21]. Diverse cell-intrinsic cascades and microenvironmental signals act synergistically to amplify the systemic senescent burden. Physiological aging functions as the primary driver, relentlessly triggering telomere attrition and progressive genomic instability [22,23]. Beyond chronological progression, these senescence trajectories are significantly modulated and accelerated by external exposures and lifestyle factors. Pathological states including obesity, metabolic dysregulation, and degenerative syndromes, together with environmental insults such as tobacco use, ethanol consumption, and pollution exposure, induce profound oxidative stress and persistent low-grade inflammation that ultimately exacerbate cellular decay [24].

The aging process extends to the immune system through immunosenescence, driving a progressive decline in immune function that reshapes the composition, density, and activity of immune organs, cells, and signaling molecules [25]. At the molecular level, immunosenescence shares several defining hallmarks with canonical cellular senescence, including cell-cycle arrest, apoptosis resistance, SASP secretion, telomere shortening, etc. Uniquely, it is also characterized by metabolic reprogramming marked by increased glycolysis, elevated Reactive oxygen species (ROS), and reduced mitochondrial function driven by persistent antigenic exposure; the aging immune system produces sustained pro-inflammatory signaling and cytokine release [26]. This chronic low-grade inflammation (inflammaging) elevates inflammatory markers across tissues and blood, posing a major risk factor for age-related morbidity and mortality [27].

**Figure 1:**
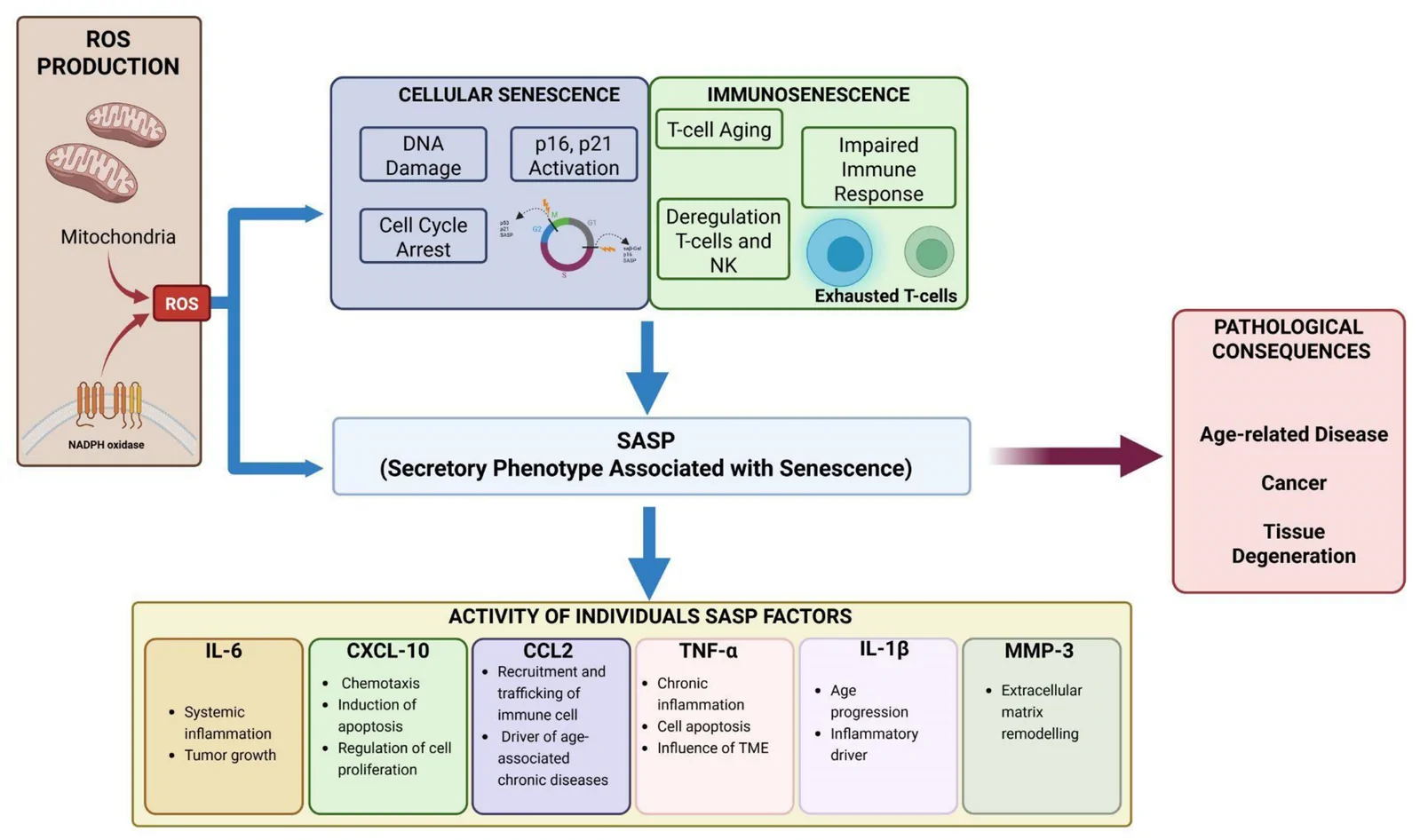
**The role of ROS in cellular senescence, immunosenescence, and SASP secretion.** Reactive oxygen species (ROS) produced by mitochondria and NADPH oxidase induce both cellular senescence (marked by DNA damage, p16 and p21 activation, and cell cycle arrest) and immunosenescence (characterized by T-cell aging, impaired immune response, deregulation of T-cells and natural killer cells, and T-cell exhaustion). These processes lead to the induction of the senescence-associated secretory phenotype (SASP). Individual SASP factors, including IL-6, CXCL-10, CCL2, TNF-α, IL-1β, and MMP-3, mediate distinct functional outcomes such as systemic inflammation, immune cell trafficking, tissue apoptosis, and extracellular matrix remodeling. Collectively, SASP secretion contributes downstream to pathological consequences including age-related diseases, cancer, and tissue degeneration. [Created in BioRender. Tommasi, S. (2026) https://BioRender.com/7k66wce].

One of the primary causes of the onset of this state of chronic systemic inflammation is the release of SASP by senescent cells. SASP promotes cellular senescence through autocrine and paracrine mechanisms, inducing senescence in adjacent cells that lack hallmarks of aging. Simultaneously, it alters innate and adaptive immune systems, leading to immunosenescence characterized by the dysregulation of signaling pathways and altered senescence and regulatory capacities within immune cell populations. Cellular senescence, immunosenescence, and inflammaging are thus functionally interconnected. Immunosenescence entails a decline in immune surveillance and responsiveness, while inflammaging perpetuates chronic low-grade inflammation through persistent immune activation. Together, these processes give rise to a feedback mechanism that exacerbates age-related pathologies (Figure 1) [28,29].

### 2.1. The ROS-Inflammaging Cycle

Reactive oxygen species (ROS) function as essential intracellular signaling molecules. However, due to their intracellular accumulation, they can react with major cellular macromolecules, including lipids, proteins, and nucleic acids, driving the progressive accumulation of molecular damage associated with aging and age-related pathologies [30]. Mitochondria and Nicotinamide Adenine Dinucleotide Phosphate (NADPH) oxidases of the NOX family represent the primary intracellular sources of endogenous ROS.

Mitochondrial ROS accounts for approximately 90% of all intracellular ROS production. This specific oxidative stress is a primary driver in the accumulation of harmful mitochondrial DNA (mtDNA) mutations, a process central to the biological mechanisms of aging. As age progresses, mitochondrial function gradually declines, leading to impaired energy production and further increasing mitochondrial dysfunction [31]. In aging, the persistent increase in ROS levels contributes to the maintenance of a pro-inflammatory state, promoting cytokine production such as IL-6 and TNF-α, while simultaneously impairing immune cell function, thus linking oxidative stress to both inflammaging and immunosenescence.

The NOX family is responsible for the production of O_2_ or H_2_O_2_ radicals through the transfer of electrons from cytoplasmic NADPH, via the catalytic NOX subunit, to molecular oxygen. The ROS generated by these enzymes play a crucial role in the activity of neutrophils and macrophages, where they contribute to effective bacterial killing and host defense. Upon recognition of endogenous or exogenous danger signals, the NADPH oxidase complex relocates and assembles at the plasma membrane, leading to the formation of the phagosome. This event triggers the generation of large quantities of highly reactive ROS, a process known as the phagocytic burst, which is highly efficient in defeating pathogens. Although the NOX isoenzymes were originally linked to ROS production in phagocytes, it is now clear that other members of the NOX family perform different regulatory functions across multiple tissues and may contribute to aging and age-related diseases [32]. Chronic, low-grade inflammation is frequently driven by the overactivation of NOX isoenzymes (like NOX2 or NOX4). This has been linked to neurodegeneration, cardiovascular disease, fibrosis, and cancer [33]. In fact, in malignant contexts, aberrant NOX enzyme activity shifts the cellular redox state toward a pro-tumorigenic imbalance through excessive ROS production. Although targeting NOX pathways offers a potent strategy for cancer therapy, current inhibitors often lack specificity. Consequently, research is shifting toward identifying isoenzyme-selective inhibitors in order to minimize off-target toxicity and improve therapeutic outcomes [34].

**Figure 2:**
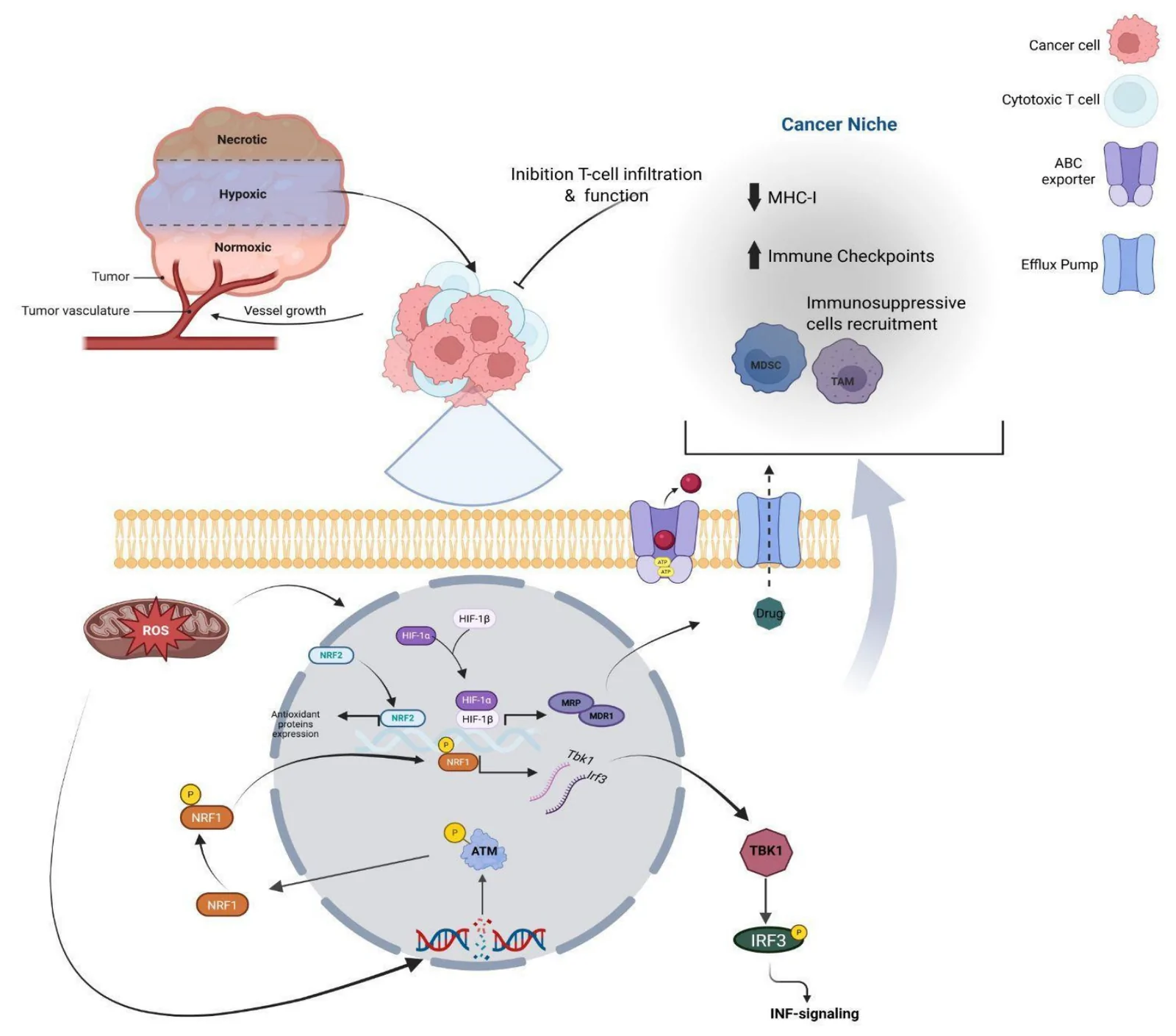
Regulation of the tumor microenvironment. Intracellular stress and genomic instability spark the liberation of ROS alongside cellular degeneration; events intrinsically linked to metabolic reprogramming. NRF1 and NRF2 transcription factor networks orchestrate antioxidant defense responses under heightened oxidative strain, whereas HIF-1α stabilization upregulates ABC efflux transporters such as MDR1 and MRPs, directly promoting multidrug resistance. Concurrently, ROS-mediated genomic instability recruits the ATM kinase to stimulate the TBK1/IRF3 signaling axis and type I interferon output, further modulating the inflammatory secretome. Coordinated with these cell-intrinsic survival cascades, signal transduction into the surrounding stroma actively reorganizes the microenvironmental landscape into an immunosuppressive niche. Neoplastic populations blunt anti-tumor immune responses by suppressing MHC-I antigen presentation, upregulating immune checkpoint ligands, and liberating chemokines that recruit MDSCs and TAMs to impair cytotoxic T-cell function. Together, these intertwined mechanisms dictate immune evasion and fuel persistent malignant progression. [Created in BioRender. Tommasi, S. (2026) https://BioRender.com/sxm2j12].

Sustained oxidative stress resulting from excessive ROS accumulation perpetually reinforces a pro-inflammatory microenvironment, driving the onset of inflammaging. This chronic inflammatory state significantly impairs anti-tumor immune surveillance, ultimately facilitating immune evasion by neoplastic populations. Heightened ROS generation triggers the activation of the transcription factor nuclear respiratory factor 1 (NRF1), a critical mediator of cellular senescence and age-associated physiological decline across diverse tissues. Mechanistically, ROS-mediated genomic instability recruits the ATM kinase, which directly phosphorylates NRF1 at Ser393. This post-translational modification robustly stimulates the NRF1–TBK1/IRF3 type I interferon signaling axis, thereby accelerating cellular senescence trajectories and amplifying the secretome burden of the SASP [35]. This vicious hypoxic-oxidative feedback loop upregulates drug efflux transporters, activates antioxidant defense networks like the transcription factor nuclear factor erythroid 2-related factor 2 (NRF2), and induces genomic instability. Consequently, cancer cells within these hypoxic pockets gain an MDR phenotype and a blunted response to therapy (Figure 2) [36]. Limited oxygen availability impairs cellular metabolism and triggers a continuous surge of ROS, which stabilizes key hypoxic signaling pathways like Hypoxia-Inducible Factor 1-alpha (HIF-1α) [37]. Understanding the balance between “primary ROS” signaling and deleterious, tumor-promoting oxidative stress is essential for designing treatments that exploit ROS vulnerability to eliminate cancer cells without causing off-target toxicity.

### 2.2. ROS-Driven Inflammasome Activation

**Figure 3:**
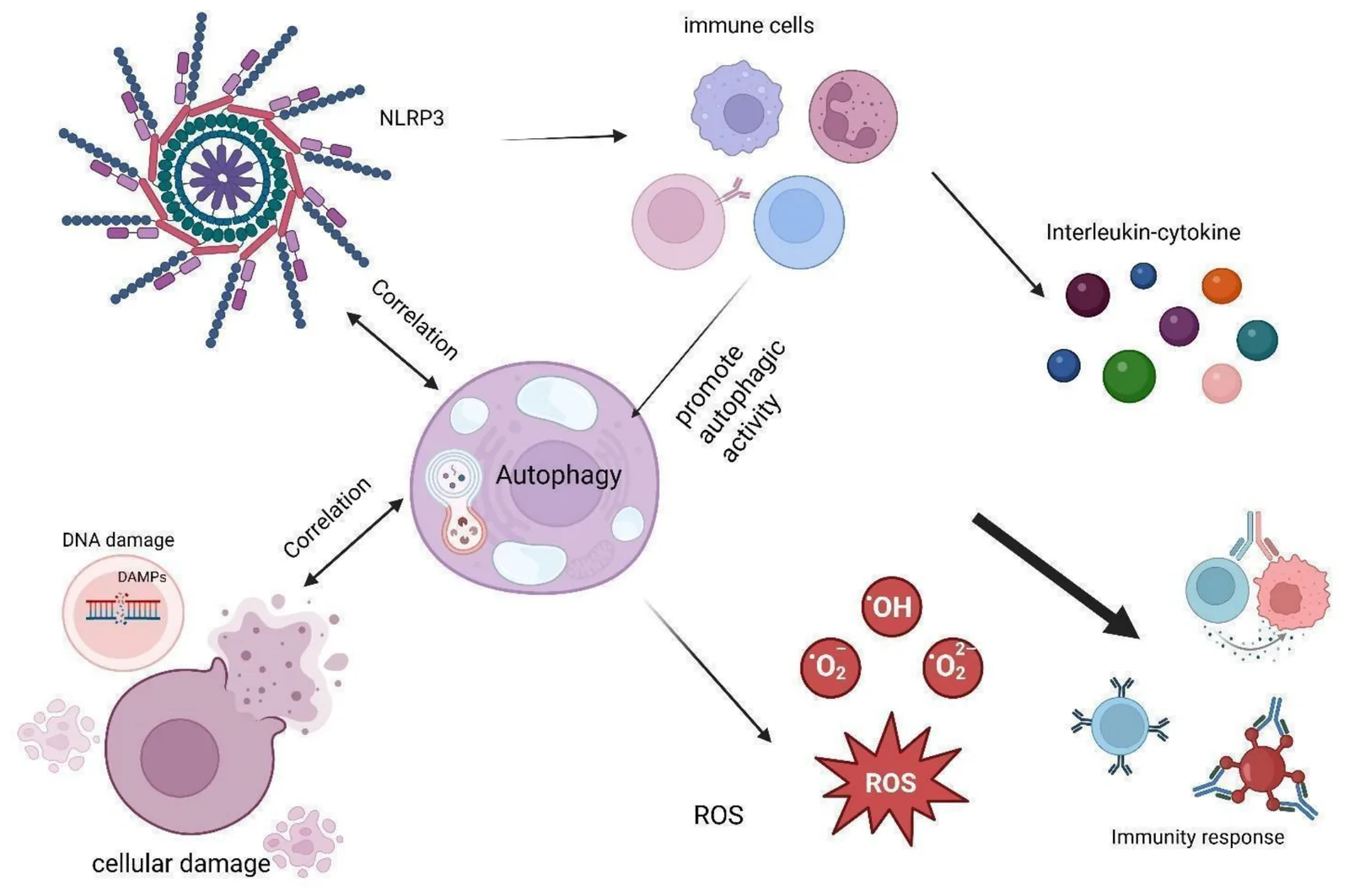
Regulation of the NLRP3 Inflammasome Architecture. Intracellular strain and genomic instability spark the liberation of damage-associated molecular patterns (DAMPs) alongside cellular degeneration, events intrinsically linked to autophagic degradation dynamics. Crucially, autophagy engages in a bidirectional interplay with the NLRP3 inflammasome network, modulating functional immune cell recruitment. Activated immune lineages directly stimulate autophagic pathways while liberating a diverse array of interleukin cytokines. Coordinated with ROS, superoxide anions, and peroxides under autophagic control, these intertwined cascades dictate downstream immune effector responses and fundamentally reorganize the microenvironmental landscape. [Created in BioRender. Tommasi, S. (2026) https://BioRender.com/uj3bu5u].

Cellular oxidative stress driven by ROS production promotes inflammasome activation. Inflammasomes are protein complexes found inside cells that build up to coordinate the body’s immune response against microbial infections and cellular damage [38]. NOD-, LRR-, and pyrin domain-containing protein 3 (NLRP3) represents one of the principal inflammasome sensors of intracellular stress signals, particularly damage-associated molecular patterns (DAMPs) (Figure 3). Together with misfolded or aggregated proteins released from destabilized lysosomes and dysfunctional mitochondria, these stress signals enhance ROS and promote NLRP3 activation [39]. Once activated, the NLRP3 inflammasome triggers the inflammatory cascade by promoting the activation of caspase-1, which processes the inactive precursors of pro-inflammatory cytokines, such as IL-1β, IL-1α, and IL-18, into their active forms, while also sustaining interaction with NF-κB [40]. Although NLRP3 exhibits low basal activity under resting conditions, the initiation of the inflammatory response requires a tightly regulated priming and oligomerization phase involving NF-κB, thereby adding multiple levels of control to this process. In substance, the NLRP3 inflammasome is a main component of the innate inflammatory response to pathogenic infection and tissue damage.

### 2.3. Autophagy, NLRP3 Activation, and the Inflammatory Microenvironment of Aging and Cancer

To support NLRP3 activity, autophagy works in tandem with the NLRP3 inflammasome to balance the production and degradation of cellular components, playing a pivotal role in healthy growth, development, and cellular maintenance. Autophagy is a quality control system ensuring the preservation of normal cellular homeostasis [31,41]. Additionally, the efficiency of autophagic degradation decreases with age, and this reduction may itself play a role in driving the aging process [42]. Alterations in autophagy regulation have significant consequences for the innate immune response, as well as for aging and age-related diseases, by modifying inflammasome activation, cytokine release, antigen presentation, and lymphocyte activity [43]. The age-associated decline in autophagic degradation contributes to the persistence of immune mediators and DAMPs, which in turn sustain a cycle of chronic inflammation. It is now well established that inflammation plays a critical role in tumorigenesis. Indeed, an inflammatory microenvironment constitutes an essential element of the tumor architecture. In recent work, low NLRP3 expression in non-small cell lung cancer (NSCLC) patients’ tissues has been associated with longer survival. supporting the pro-tumorigenic role of NLRP3. By driving the maturation and release of pro-inflammatory cytokines IL-1β and IL-18, it amplifies both local and systemic inflammation while promoting tumor growth and metastatic progression [44]. This mechanism has also been underlined in “in vivo” and “in vitro” models of primary mouse peritoneal macrophages and chronic colitis, in which the pharmacological support of inflammation activated the caspase-11-dependent noncanonical NLRP3 inflammasome and the subsequent upregulation of IL-1b and IL-18 [45]. Beyond promoting proliferation, NLRP3 plays a vital role in the TME, particularly in pancreatic ductal adenocarcinoma (PDAC), where, in an “in vivo” model, it acts within macrophages as a key driver of immune evasion. By releasing IL-10, these macrophages skew CD4+ T cell differentiation toward pro-tumorigenic populations (Th2, Th17, and Tregs) while simultaneously inhibiting protective Th1 responses and CD8+ cytotoxic T cell activation. Genetic deletion or pharmacological blockade of the NLRP3 pathway reverses this immune suppression, converting the pancreatic TME into an immunogenic state [46]. Experimental evidence has also demonstrated significant dysregulation of NLRP3 inflammasome activation in chronic myelomonocytic leukemia (CMML) cohorts, showing a robust positive correlation with disease severity [47]. This suggests that the aging process induces a heightened cellular sensitivity to NLRP3 activation. Such primed inflammatory responses likely provide a mechanistic basis for the systemic inflammation and immune dysregulation frequently observed in older populations.

A part of the literature reports an anti-tumorigenic role of the NLRP3 inflammasome. Research using mouse models of acute colitis and colitis-associated cancer shows that the inflammasome serves a protective function in the gut, dampening both tissue inflammation and tumorigenesis. Deletion of the inflammasome components ASC (PYCARD) and caspase-1 significantly worsens disease severity, morbidity, tissue damage, and tumor growth in mice, due to impaired local production of IL-1β and IL-18. Specific expression experiments demonstrate that NLRP3 protects against tumor formation through its action in hematopoietic immune cells rather than in intestinal epithelial or stromal compartments [48]. In diffuse large B-cell lymphoma cell lines, NLRP3 inflammasome activation upregulates PD-L1 expression and decreases the population of cytotoxic T cells. Conversely, “in vivo” blockade of the NLRP3 inflammasome suppresses lymphoma growth and enhances anti-tumor immunity by downregulating PD-L1 within the TME [49]. Taken together, inflammasomes exert a dual, context-dependent role in cancer. They can either promote tumor progression by driving inflammation, stemness, metastasis, and immune evasion, or suppress tumors by activating anti-tumor immunity, tumor suppressors, and pyroptosis. Their final impact depends on the specific cell type, tissue context, and TME.

## 3. Pro-Inflammatory Mediator Overview

Pro-inflammatory mediators are a group of secreted molecules produced by immune and non-immune cells in response to tissue damage, infection, or metabolic stress, and orchestrate the initial inflammatory response. While essential for acute healing and defense, their sustained or dysregulated release drives persistent tissue damage and underlies the pathogenesis of numerous chronic disorders, including cardiovascular diseases, autoimmune conditions, and cancer progression [50,51].

### 3.1. Interleukin-Family

#### 3.1.1. Interleukin-1 (IL-1)

**Figure 4:**
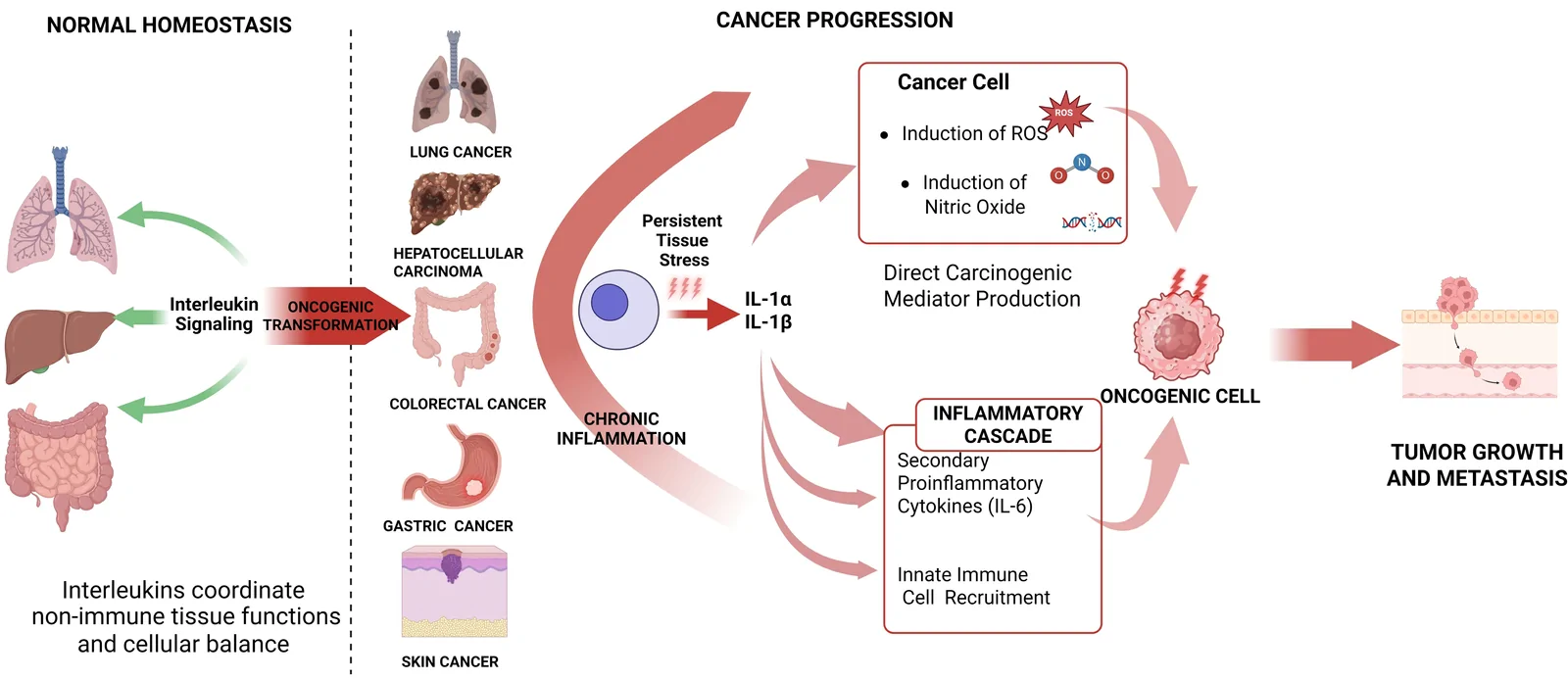
Interleukin-mediated regulation across physiological steady-states and neoplastic progression. Physiological homeostasis (**left**): In healthy microenvironments, interleukin cascades sustain tissue architecture and systemic balance across epithelial compartments (such as pulmonary, hepatic, and gastrointestinal tracts). Smoldering inflammation and malignant remodeling (**right**): Unresolved tissue damage sparks persistent inflammatory signaling that forces pre-malignant transformation, accelerating oncogenesis and promoting local invasion alongside metastatic dissemination. [Created in BioRender. Tommasi, S. (2026) https://BioRender.com/tiv89tc].

As master orchestrators of the stress-induced inflammatory cascade, IL-1α and IL-1β (collectively IL-1) and IL-18 dictate cellular stress responses. Their activation follows distinct molecular pathways: while caspase-1 cleaves IL-1β and IL-18 in their mature forms, calpain processes IL-1α [52,53]. Once active, all three engage the IL-1 receptor (IL-1R), an interaction regulated by the native inhibitor IL-1 receptor antagonist (IL-1Rα). Intriguingly, aging unravels a striking paradox within the system. In fact, circulating levels of IL-1β remain surprisingly stable with age progression, even though IL-1Rα spikes sharply with age. This age-related elevation in IL-1Rα correlates with increased comorbidity, age-related diseases, and higher mortality [54]. IL-18, a pro-inflammatory cytokine related to IL-1, signals through a receptor complex composed of the IL-18 receptor α (IL-18Rα) and β (IL-18Rβ) chains and stimulates the production of Interferon gamma (IFN-γ), which is crucial for host defense against infections [55,56]. IL-18 collaborates with IL-1 to orchestrate systemic immune responses and inflammation linked to the aging process [31]. Because IL-1α and IL-1β are exceptionally potent inflammatory drivers, they typically circulate at near-undetectable levels, policed by decoy receptors and soluble antagonists [57]. As the IL-1 family constitutes the primary triggers of the stress-induced cascade, their regulation in the aging context appears as an intriguing paradox. Far from being a successful defense mechanism, this elevated IL-1Rα likely serves as a biological alarm, a “biomarker of resistance” reflecting a chronic, underlying fire the body strongly, yet unsuccessfully, tries to extinguish [58,59]. Rather than shielding the host, this rise in antagonists signals a state of smoldering, persistent immune activation tightly tied to the metabolic decline of aging [60]. Beyond maintaining tissue homeostasis, interleukin signaling becomes a pathological double-edged sword after malignant transformation, driving tumor expansion and metastatic spread across a wide range of cancers, from gastric and respiratory malignancies to skin and liver carcinomas [61]. Within this inflammatory microenvironment, IL-1α and IL-1β play a dual role: they directly induce carcinogenic mediators like nitric oxide (NO) and ROS while triggering downstream waves of pro-inflammatory cytokines, such as Interleukin-6 (IL-6) [62,63] (Figure 4). This IL-1-driven cascade recruits innate immune cells, fueling a self- sustaining loop of tissue damage and malignant progression.

#### 3.1.2. Interleukin-6 (IL-6)

Originally characterized as a B-cell growth factor, interleukin-6 (IL-6) was subsequently established as a pivotal pro-inflammatory mediator of the innate immune response, predominantly synthesized by monocytes and activated macrophages. Beyond classic immune lineages, IL-6 expression and secretion occur across diverse cellular populations, including fibroblasts, endothelial cells, keratinocytes, mesangial cells, and various neoplastic lineages [57]. This pleiotropic cytokine functions as a master regulator orchestrating the acute-phase cascade, metabolic homeostasis, the transition from innate to adaptive immunity, and the pathogenesis of chronic inflammatory disorders. Although circulating levels remain minimal under physiological baseline conditions, systemic IL-6 concentrations experience a sustained age-associated elevation, serving as a robust surrogate indicator of systemic inflammaging and biological frailty [64]. Mechanistically, persistent genomic stress recruits ATM, NBS1, and CHK2 kinases to directly stimulate IL-6 release from senescent populations [65,66]. Once secreted, this factor operates through self-sustaining autocrine and paracrine signaling loops to reinforce and propagate cellular senescence across neighboring tissues [67]. Herbstein and collaborators uncovered a novel, non-canonical intracrine pathway through which IL-6 regulates senescence from within the cell. The authors demonstrate that a fraction of synthesized IL-6 bypasses the classical secretory pathway, remaining inside the cell to interact with intracellular IL-6 receptors (IL-6R) within anterograde trafficking compartments. This internal ligand-receptor engagement triggers a non-canonical activation of the cGAS-STING pathway, leading to the activation of NFκB. This establishes a self-sustaining feedback loop required to maintain stable cell-autonomous senescence [68].

By saturating the circulation, this secretome destabilizes the precise metabolic and immune homeostasis that IL-6 normally maintains. Such a systemic breakdown in communication could transform IL-6 into a dual-threat: acting as both a sentinel marker of biological aging and a probable participant in the progression toward chronic disease and mortality. The role of the innate immune system in oncology is famously double-edged. In the interplay between inflammation and cancer, pro-inflammatory mediators modulate the inflammatory response, either suppressing tumor progression through acute immune surveillance or promoting tumor growth and metastasis when signaling becomes chronic [69,70]. In the aging individual, this balance is frequently tipped toward the latter. Mediators like IL-1 and IL-6 cease to act as alarms for repair and instead begin to remodel the tissue microenvironment [71]. This shift effectively recruits myeloid-derived suppressor cells (MDSCs), and appears to promote angiogenesis, transforming a defensive immune posture into a permissive, pro-tumorigenic landscape [72]. Abnormal levels of cytokines and chemokines significantly accelerate breast cancer (BC) progression in postmenopausal women. They achieve this by fueling cancer cell development and signaling, as well as signaling to other cells in the TME to initiate the invasion process [73]. The secretome of tumor-associated immune cells varies by cancer type; however, these cells consistently release cytokines and chemokines that facilitate both malignant proliferation and immune evasion [74]. In summary, while acute inflammation is a vital, self-limiting defense mechanism necessary for tissue repair and pathogen eradication, its failure to resolve can lead to a detrimental state of chronic, subclinical inflammation. This “dormant” inflammatory environment is a significant driver of age-related pathologies, most notably facilitating malignant progression. The transition from a protective to a pro-tumorigenic state is heavily influenced by the interleukin profile in older patients. In the aging population, dysregulated interleukin signaling not only fuels cancer cell development and invasion but also impairs the efficacy of the immune response. Consequently, understanding the specific mechanisms by which these interleukins orchestrate the TME is essential for developing targeted preventative and therapeutic strategies tailored to the unique physiological landscape of older adults [75].

### 3.2. Tumor Necrosis Factor-Alpha (TNFα)

Synthesized predominantly by monocyte-macrophage lineages and dendritic populations, tumor necrosis factor-alpha (TNF-α) functions as a crucial pro-inflammatory mediator that preserves local tissue homeostasis; however, its systemic dissemination determines profound pathological cascade effects [76]. It is initially synthesized as a non-glycosylated type II transmembrane protein, which can subsequently be cleaved to generate its soluble form [77]. TNF-α plays a central role in mediating local inflammation and can contribute to tumor-associated vascular thrombosis and regulate the function of immune system cells [78,79]. TNF-α promotes its biological effects through binding to two distinct receptors, TNF receptor I (TNFRI) and TNF receptor II (TNFRII). TNFRI, also known as CD120a, is encoded by the TNFRSF1A gene and represents the main mediator of TNF-α-induced cytotoxic signaling [80,81]. The interaction between TNF-α and its receptors initiates multiple intracellular signaling pathways responsible for its downstream effects. In particular, binding of TNF-α to TNFRI promotes receptor trimerization and recruitment of adaptor proteins such as TRADD (TNF receptor-associated death domain). TRADD subsequently interacts with FADD (Fas-associated death domain), leading to the activation of caspase 8. This event triggers a caspase cascade that ultimately culminates in programmed cell death [81,82]. Research shows that older adults, including centenarians and octogenarians with atherosclerosis, often have elevated levels of TNF-α. High levels of this cellular aging marker are strongly linked to an increased risk of death [31]. Dysregulated TNF-α signaling has also been implicated in tumor cell proliferation, survival, invasion, angiogenesis, and metastasis. Recent studies demonstrate that triggering the GPR68-ATF4 pathway leads to a significant increase in IL-6 and TNF-α production. This process allows the tumor to bypass immune detection, ultimately accelerating its growth and cell survival [83,84]. In older male cancer survivors, chronic fatigue was significantly associated with higher levels of IL-6 and TNF-α. As a pivotal pro-inflammatory cytokine, TNF-α heavily impacts both metabolic and neural function [85]. By triggering NF-kB signaling, boosting ROS, and disrupting mitochondrial ATP production, TNF-α drives cellular energy failure, ultimately leading to cancer-related fatigue [86]. Clinical evaluation of anti-TNF-α interventions within elderly rheumatoid arthritis cohorts diagnosed with colorectal, pulmonary, or prostatic malignancies revealed no adverse impact on overall survival (OS) relative to standard synthetic disease-modifying antirheumatic regimens, establishing the therapeutic viability of these biologic agents in aged demographics [87]. Consequently, precise and dynamic profiling of systemic TNF-α concentrations provides an actionable tool for oncologic stratification and monitoring [88]. Nevertheless, TNF-α signaling exhibits complex, context-dependent effects in mammary carcinoma. In experimental models of breast cancer, pro-inflammatory cascades mediated by TNF-α, IL-1β, and IL-6 restrict neoplastic proliferation by uncoupling downstream IGF-I receptor signal transduction independently of receptor tyrosine kinase inhibition. These cytokines enforce G0/G1 cell-cycle arrest, preventing entry into the S phase [89]. Recombinant murine TNF-α demonstrates potent anti-tumor activity across syngeneic and xenograft murine models via combined direct cytotoxicity and stromal remodeling [90]. Moreover, engineered oncolytic adenoviral vectors co-expressing TNF-α and IL-2 effectively reverse immune checkpoint resistance by stimulating tumor-infiltrating T-cell expansion and activation. Despite these promising preclinical anti-tumor mechanisms, clinical translation was initially limited by systemic toxicity and off-target adverse effects [91,92]. This functional dichotomy is governed by microenvironmental architecture, local growth factor availability, and host-level inflammatory tone, which are further exacerbated by age-associated baseline cytokine elevations.

### 3.3. The Chemokine Interferon-γ Inducible Protein 10 kDa (CXCL10)

The chemokine interferon-γ–inducible protein of 10 kDa (CXCL10) is a member of the CXC chemokine family, which plays a key role in chemotaxis [93], induction of apoptosis [94], regulation of cell proliferation [95,96], and mediation of angiostatic activity [97]. Usually, CXCL10, together with other CXC chemokines, induces its effects through binding to G protein-coupled receptors, primarily C-X-C motif chemokine receptor 3 (CXCR3), thereby activating downstream signaling pathways that regulate different biological and physiological processes. Dysregulated CXCL10 expression has been associated with a wide range of human disorders, including infectious diseases [98], chronic inflammatory conditions [99,100], immune dysfunction [101], tumor development, metastasis, and disease dissemination [102,103]. Within these malignant contexts, CXCL10 serves as a pleiotropic regulator of cellular growth dynamics. Quantitative intratumoral CXCL10 abundance acts as an essential determinant of microenvironmental status, where robust infiltration of CXCL10-expressing populations correlates with favorable recurrence-free and overall patient survival across diverse solid carcinomas [104]. Crucially, investigations reveal that anti-tumor effector cascades elicited by distinct treatment regimens are functionally dependent on local CXCL9 and CXCL10 levels. In addition, elevated CXCL10 secretome density fosters an immunologically active niche by promoting targeted recruitment of cytotoxic CD8+ T lymphocytes and CD20+ B cell lineages, thereby serving as an independent prognostic marker for extended progression-free survival [105]. In an in vivo murine model of head and neck squamous cell carcinoma, intratumoral CXCL10 reprograms immunosuppressive head and neck tumors by enhancing the recruitment, survival, and antitumoral activity of CD8+T, CD4 T, and NK cells while simultaneously stunting angiogenesis. CXCL10 also reinvigorates exhausted CD8+ T cells and promotes T cell priming within tumor-draining lymph nodes, establishing a continuous feedback loop of effector cell trafficking into the tumor [106]. These recruited immune cells drive anti-tumor responses through enhanced surveillance, efficient antigen presentation, and direct tumor cell lysis. In parallel, CXCL10 establishes a pro-inflammatory environment that further amplifies immune activation. In cervical carcinoma, CXCL10 suppresses tumor progression by inhibiting microvessel formation, reducing proliferating cell nuclear antigen (PCNA) expression, and downregulating the High-Risk human papillomavirus (HPV) oncoproteins E6 and E7, at least in part through the promotion of apoptosis [107]. Other anti-tumor effect studies through angiostatic action are conducted in xenograft models of lymphoma [108,109], squamous cell carcinoma [110], and lung adenocarcinoma [111,112]. Elevated levels of CXCL10 expression have been reported to correlate inversely with tumor growth, concomitantly leading to a marked reduction in tumor-associated angiogenesis. Importantly, these antitumor effects appear to occur independently of T cells, macrophages, and natural killer (NK) cells, suggesting a direct impact on tumor and endothelial compartments [108,113]. CXCL10 has also been shown to counteract basic fibroblast growth factor (bFGF)-mediated angiogenesis in advanced uterine endometrial cancer. Additionally, CXCL10 exerts potent immunogenic antitumor effects. Accumulating evidence indicates that CXCL10 contributes to antitumor immunity through its immunomodulatory functions. Specifically, it facilitates the recruitment of immature antigen-presenting cells, dendritic cells, and early activated T cells to the tumor site in murine glioma and melanoma models [114].

Conversely, in tumor cells, CXCL10 has been observed to colocalize with the proliferation marker cytokeratin 17 (CK17), a protein known to be associated with cell cycle progression. This spatial association suggests a potential involvement of CXCL10 in cell cycle-dependent proliferative signaling [95]. In colorectal adenocarcinoma (CRC), CXCL10 serves as a critical indicator of TME status. Although IFN-gamma primarily triggers CXCL10 to stimulate an anti-tumor immune response, autocrine signaling within the tumor cells paradoxically enhances their growth and metastatic potential. In human BC cell lines, including MDA-MB-435 and MCF-7, oncogenic Ras has been shown to induce CXCL10 overexpression via the Raf and phosphatidylinositol 3-kinase (PI3K) signaling pathways. Increased CXCL10 levels may subsequently promote autocrine activation of CXCR3 while downregulating the CXCR3-B isoform, thereby favoring tumor cell proliferation and disease progression [115,116,117]. CXCL10 regulation within the TME is complex and multi-layered, with its expression and activity jointly controlled by transcriptional drivers, epigenetic modifications, metabolic reprogramming, and metabolite-mediated signaling. Mechanistically, stroma-derived CXCL10 binds CXCR3 on tumor cells to trigger downstream MAPK/ERK and PI3K/Akt signaling cascades. This pathway activation induces cytoskeletal remodeling, prolongs cell survival, and drives directional migration, enabling tumor cells to breach the extracellular matrix and intravasate into the bloodstream. Furthermore, CXCL10 remodels the metastatic niche by blunting cytotoxic immune responses and driving M2 macrophage polarization, thereby establishing a permissive environment for tumor seeding [118].

As a result of this regulatory diversity, CXCL10 exerts dual, context-dependent effects on cell growth, acting as either a proliferative or anti-proliferative signal [119]. On the one hand, the anti-proliferative effects of CXCL10 are primarily mediated by a specific receptor isoform, CXCR3-B, which possesses growth-inhibitory properties; on the other hand, the proliferative isoform CXCR3-A promotes cell proliferation [120]. A recent study reveals that aging in both human and mouse breast tumor cells leads to diminished production of the T cell-attracting chemokines CXCL9 and CXCL10. This deficit fosters an immunosuppressive microenvironment that drives tumor progression, establishing a direct causal link between age-related immune dysfunction and accelerated tumor growth [121]. In a CRC model, soluble biomarkers, including CXCL10, showed strong promise as detection biomarkers for elderly CRC patients [122]. Although these findings mark significant progress in identifying soluble indicators of immune senescence, clarifying their broader impact on the aging immune system will be essential for leveraging them into novel cancer treatments.

### 3.4. The Chemokine Ligand CCL2

The chemokine ligand CCL2, also referred to as monocyte chemoattractant protein-1 (MCP-1), was first identified and purified in 1989 from the supernatants of cultured peripheral blood mononuclear cells and tumor cell lines [123]. CCL2 modulates the recruitment and trafficking of multiple immune cell types, such as monocytes, memory T cells, and NK cells, highlighting its central function in shaping the immune microenvironment [124]. As one of the earliest characterized members of the CC chemokine family, CCL2 primarily exerts its biological functions through interaction with its receptor, CCR2 [125]. Accumulating evidence indicates that the CCL2-CCR2 signaling axis contributes to multiple aspects of tumor biology, including pathological angiogenesis, tumor cell survival, invasion, and the recruitment of immunosuppressive cell populations within the TME [126,127]. The CCL2-CCR2 signaling axis, essential for monocyte and macrophage recruitment, has been increasingly linked to the pathophysiology of aging [128]. Recent studies have expanded its role from simple inflammatory mediation to a key driver of age-associated chronic diseases, including neurodegeneration and cancer development [129,130]. Given its prominent role in driving tumor cell proliferation and chemoresistance, the CCL2-CCR2 signaling axis represents a highly attractive therapeutic target, with novel inhibitors demonstrating promising antitumor efficacy in recent preclinical and clinical studies [131,132]. Overexpressed across a wide spectrum of malignancies, the CCL2-CCR2 axis has been consistently identified as an independent risk factor for advanced clinical stage, tumor recurrence, and reduced overall or disease-free survival. High serum levels of CCL2 correlate strongly with tumor aggressiveness and poor prognosis in major cancer types, including breast [133], prostate [134], lung [135], hepatocellular [136], pancreatic [137], and CRC [123], as well as in numerous other solid tumors and hematological malignancies [126,138,139,140]. Overexpression of CCL2 and/or its receptor CCR2 has also been significantly correlated with reduced survival in nasopharyngeal carcinoma, cervical cancer, ovarian cancer, hepatocellular carcinoma, and clear-cell renal cell carcinoma, where the CCL2-CCR2 axis may serve as an independent prognostic factor. Additional evidence supports the prognostic relevance of CCL2 in bladder, esophageal, thyroid, and liver cancers, as well as in osteosarcoma, salivary adenoid cystic carcinoma, and glioma, where CCL2 overexpression is frequently associated with tumor progression, recurrence, advanced clinical stage, and decreased OS [126,140]. CCL2 promotes tumor cell growth and proliferation through multiple mechanisms. Evidence indicates that CCL2 promotes the survival of prostate cancer PC-3 cells by inhibiting autophagic cell death, particularly under serum-free conditions, via activation of the PI3K/AKT signaling cascade [141,142]. Furthermore, by suppressing docetaxel-induced caspase-3 activation, CCL2 attenuates apoptosis and supports tumor cell proliferation. Mechanistically, this process is linked to PI3K/AKT pathway activation, with CCL2 promoting AKT phosphorylation at Ser473 [143]. CCL2/CCR2 axis selectively recruits monocytes and immunosuppressive macrophages. This ‘chemokine swap’ could effectively flip the microenvironment from an immune-active state to one that shields tumor cells from surveillance, providing a mechanistic link between chronological aging and accelerated tumor progression [144]. The transition of the CCL2-CCR2 axis from a mediator of acute inflammation to a driver of chronic, age-related pathology highlights the complexity of ‘inflammaging’ in cancer [145,146]. In younger models, CCL2-mediated recruitment typically may aid in pathogen clearance; however, in the aged context, this signaling appears hijacked. When coupled with the age-associated defects in T-cell attraction, the overactivation of CCL2-CCR2 likely reinforces a pro-tumorigenic niche [147,148]. The convergence of immunosenescence and TME creates a unique clinical challenge in oncology. As the immune system ages, it undergoes a shift toward chronic, low-grade inflammation, which fundamentally alters how the body responds to malignancy. This suggests that in older populations, the CCL2-CCR2 signaling axis is not merely an active pathway but a dominant driver of cancer progression. Thus, the therapeutic targeting of CCR2 may be particularly effective in older patient populations where this axis dominates the immune landscape.

### 3.5. Matrix Metalloproteinase MMP3

MMPs play critical roles in diverse pathological conditions, ranging from cancer invasion and metastasis to cardiovascular and inflammatory diseases [149,150,151]. In oncology, MMPs are implicated in tumor initiation, progression, and metastatic dissemination, and their activity is tightly linked to immune system modulation. The MMPs primarily function by degrading components of the extracellular matrix (ECM) and have been known to promote cancer invasion and metastasis. As ECM is essential for maintaining tissue architecture and regulating cell proliferation, differentiation, migration, and intercellular signaling, its remodeling by MMPs has profound implications for both physiological and pathological processes. The activity of MMPs is tightly controlled at multiple levels, including transcriptional regulation, inhibition by endogenous tissue inhibitors of metalloproteinases (TIMPs), and proteolytic activation of their inactive proenzyme forms [152]. Matrix metalloproteinase-3 (MMP-3) is produced as a latent precursor (zymogen) composed of three distinct domains: the N-terminal pro-domain, the catalytic domain, and the hemopexin-like C-terminal domain. The pro-domain contains a conserved cysteine switch sequence that interacts with the catalytic zinc ion, thereby maintaining the enzyme in an inactive conformation and preventing premature proteolytic activity. Enzymatic activation occurs following disruption or removal of the pro-domain, which exposes the catalytic site and enables substrate cleavage [153,154,155]. Once activated, MMP-3 degrades multiple extracellular matrix components, promoting tissue remodeling and facilitating processes such as cell migration and tumor progression [155,156]. In recent studies, the discovery of high levels of MMP-3 is associated with a poor chemotherapy response in various solid tumors, suggesting that targeting could improve therapeutic outcomes [156]. MMPs play a complex, context-dependent role in tumor vessel formation, dynamically shaping the vascular landscape based on their expression timing and local substrate availability. Beyond driving vessel growth, MMPs act as key negative regulators of angiogenesis by generating anti-angiogenic degradation fragments. Through the targeted cleavage of type IV collagen, type XVIII collagen, plasminogen, and perlecan, MMPs liberate tumstatin, endostatin, angiostatin, and endorepellin, respectively, to restrict vascular sprouting and maintain the angiogenic balance, controlling cancer development for which new blood vessels are necessary [157,158].

Although context-dependent effects have been described, MMP-3 appears to be more commonly associated with tumor-promoting functions. Upregulated MMP-3 expression is shared across a wide spectrum of malignancies, including breast, lung, colorectal, and pancreatic carcinomas, where it consistently correlates with accelerated tumor progression and unfavorable patient prognosis [159]. As tumor cells lose their structural integrity and functional geometry, MMP-3 production typically surges. Interestingly, while high MMP-3 in the elderly often stems from immune dysregulation and senescence, relatively high levels are also observed in younger cancer patients [160]. Consequently, while MMP3 may not serve as a solo age-specific diagnostic marker, its interaction with other biomarkers could offer valuable insight into the evolving TME across different age groups. MMP-3 acts as a dual-edged regulator that facilitates tumor progression and inflammation by dismantling the body’s structural scaffolds. A critical driver of this activity is biological aging, where senescence and immune dysregulation in older populations trigger a surge in MMP-3 levels. This age-related degradation of tissue integrity creates a microenvironment highly conducive to cancer. Consequently, while MMP-3 affects patients of all ages, its intensification during the aging process makes it a vital target for improving therapeutic outcomes in age-related diseases and solid tumors.

## 4. Metabolic SASP

Expanding beyond the traditional secretion of cytokines and proteases, senescent populations undergo a radical metabolic transformation, resulting in the liberation of a specialized array of bioactive small molecules known as the “metabolic SASP.” This unique metabolomic profile serves as a functional mirror of systemic cellular decay, involving mitochondrial collapse, shifted lipid processing, and a transition toward hyper-glycolytic states. Far from being inert metabolic debris, these SASP components, including extracellular lipids, nucleotides, and short-chain carboxylic acids, operate as aggressive signaling mediators.

Metabolites produced by senescent cells modulate gene expression through targeted histone acetylation across SASP loci. This mechanistic axis uncovers a mitochondrial-epigenetic communication loop that directly sustains the secretome burden. Senescent cell populations undergo deep metabolic reconfiguration marked by enhanced mitochondrial pyruvate–citrate–acetyl-CoA transport. The resulting expansion of the nucleocytoplasmic acetyl-CoA pool provides the requisite molecular substrate for localized histone acetylation, enforcing an open, transcriptionally active chromatin landscape at SASP gene promoters. Functional execution of this epigenetic remodeling relies strictly on the mitochondrial citrate carrier SLC25A1 and cytosolic ATP-citrate lyase (ACLY) activity [161].

Moreover, metabolic SASP actively remodels the local tissue landscape, compromising the structural geometry of adjacent healthy cells and reinforcing the aging phenotype through persistent paracrine and autocrine stimulation [162]. A pivotal element of this metabolic signature is extracellular lactate, which functions as a mechanistic engine driving cellular senescence and chronic systemic inflammation. The profound metabolic reprogramming observed in senescent cells is frequently orchestrated by enzymes such as pyruvate dehydrogenase kinase 4 (PDK4), which redirects cellular energy toward aerobic glycolysis. This metabolic route triggers a substantial accumulation of extracellular lactate [163]. Rather than acting as a simple waste, this lactate serves as a potent trigger for the production of ROS via NOX1, thereby deepening DNA damage and amplifying the inflammatory secretome. Furthermore, lactate conditions the epigenetic environment through histone lactylation (notably the H4K12la mark), which impairs autophagy and accelerates tissue degradation, as seen in osteoarthritic cartilage. In hepatocellular carcinoma (HCC), LDHA-driven lactylation of histone H2B at K58 on N-Myc Downstream Regulated 1 (NDRG1) facilitates the emergence of senescence-resistant malignant lineages [164]. Taken together, these observations delineate a pathological feedback loop where glycolysis-derived metabolites sustain the senescent state, acting as active participants in the progression of various age-associated human pathologies [165,166].

Extensive investigations have also highlighted the heightened lipid synthesis characteristic of senescent cells [167]. Specifically, these populations show a significant overactivation of cyclooxygenase-2 (COX2), which fuels the secretion of pro-inflammatory oxylipins and eicosanoids, such as Prostaglandin E2 (PGE2). These findings are supported by evidence that the transgenic expression of COX-2 is sufficient to trigger a premature senescence phenotype in murine models [168]. These bioactive metabolites permeate the surrounding microenvironment, sustaining a chronic state of inflammaging through a self-perpetuating cycle involving EP2 and EP4 prostaglandin receptors [169,170]. In tissue-resident macrophages (TRMs), PGE2 activation of the EP2/cyclin AMP/protein kinase A axis drives the progressive accumulation of aged neutrophils. In detail, the EP2 signal impairs integrin-mediated recognition of senescent neutrophil surface ligands, effectively impairing phagolysosomal clearance and engulfment. This failure of macrophage-mediated surveillance permits non-cleared neutrophils to persist, undergo spontaneous degranulation, and unleash paracrine cellular stress upon adjacent parenchymal compartments, thereby accelerating organ-level physiological decline [171].

In addition, the progressive accumulation of senescent populations is characterized by a significant surge in the PGD2 metabolites, including 15-deoxy-Δ12,14-prostaglandin J2 (15d-PGJ2). This lipid mediator directly engages the RAS signal transduction network, triggering downstream ERK and p53 cascades. This signaling axis establishes a feedback loop that permanently enforces growth arrest while continuously amplifying the secretome burden of pro-inflammatory SASP factors [167,172]. In Table 1, we summarize the categorization and the biological roles of SASP components.

## 5. Therapeutic Prospectives

Integrating aging and senescence-associated biomarkers into standard therapeutic paradigms provides vital complementary insights for personalized care. While many of these molecular markers, such as SASP factors, are relatively novel and undergoing clinical trial validation, they already show promise in predicting biological frailty, treatment response, and therapy-induced toxicity. Evaluating these indicators allows clinicians to stratify therapy-induced senescence and systemic inflammaging, ultimately guiding more tailored treatment decisions and sequential interventions.

Experimental data demonstrate how circulating biomarkers of cellular senescence predict mortality beyond traditional risk factors [172]. Historically, quantifying this systemic senescence burden in clinical settings has been hindered by the lack of single, high-specificity biomarkers. To overcome these limitations, recent paradigms have shifted toward artificial intelligence-driven multi-proteomic modeling. Notably, the integration of large-scale proteomic datasets, such as the UK Biobank Pharma Proteomics Project (UKB-PPP), with advanced neural network architectures, specifically Guided Autoencoders with Transformers (GAET), has enabled the formulation of a standardized, blood-based “SASP Score.” By mapping the non-linear interactions within the circulating SASP landscape rather than evaluating isolated proteins, this deep-learning-derived biomarker signature has demonstrated powerful, age-independent predictive capacity for all-cause mortality, neurodegenerative diseases, and chronic cardiovascular conditions. Crucially, the longitudinal dynamism of this score in response to lifestyle interventions underscores its utility not only as a robust prognostic tool but also as a non-invasive surrogate endpoint to monitor the efficacy of emerging senolytic and senostatic therapies in clinical trials [173].

### 5.1. Senolytic and Senomorphic Molecules

One of the first clinical trials was conducted on fourteen patients with stable idiopathic pulmonary fibrosis (IPF), a progressive, fatal cellular senescence-associated disease, demonstrating that the senolytic combination of dasatinib plus quercetin might alleviate physical dysfunction [174,175]. A recent randomized placebo-controlled pilot trial analyzed the adverse events of the same senolytic combination in an IPF cohort of patients over 50 years, showing feasibility and good tolerance, despite the small sample size limitation [176]. A Phase II clinical evaluation in high-risk oncology patients demonstrated that pharmacological target inhibition of extracellular protein disulfide isomerase significantly attenuated circulating coagulation biomarkers and thrombotic activity without increasing bleeding risk, supporting PDI as a safe and novel antithrombotic target [177,178]. Phase II trial assessed dasatinib and quercetin combined with cyclophosphamide, fludarabine, and CAR-T cell therapy in relapsed/refractory multiple myeloma to define their involvement in the support of traditional therapy [179].

Everolimus, an oral mTOR pathway inhibitor with recognized senomorphic and immunosuppressive properties, remains under active investigation across numerous clinical trials evaluating its efficacy in diverse oncologic disorders [180,181]. Retrospective PAM50 subtyping from the BOLERO-2 cohort revealed that everolimus provides clinical benefit across intrinsic subtypes in HER2-enriched disease, highlighting the need for tailored combinatorial strategies in non-luminal phenotypes [182,183]. In a randomized phase II trial for newly diagnosed glioblastoma, adding everolimus to standard chemoradiation failed to improve progression-free survival and increased treatment-related toxicities. While median survival with everolimus matched historical data, it was inferior to that of the concurrent control group [184,185]. Recently, a randomized Phase II trial evaluating the efficacy of everolimus plus lenvatinib in metastatic bone sarcomas, incorporating longitudinal tissue biopsies and blood sampling to identify predictive biomarkers of response [186], is ongoing.

### 5.2. NOX-Selective Inhibitors

Pharmacological inhibition of NADPH oxidases (NOXs), key drivers of oncogenesis closely linked to tissue aging and cellular senescence, represents a promising strategy to target the TME. Setanaxib, a first-in-class, orally bioavailable dual NOX1/NOX4 inhibitor, is currently under clinical evaluation. Its efficacy in cancer management was demonstrated in a Phase 2 trial evaluating setanaxib plus pembrolizumab versus pembrolizumab plus placebo in patients with recurrent or metastatic squamous cell carcinoma of the head and neck. The setanaxib combination significantly improved progression-free survival and provided an OS benefit at 6 and 9 months compared to pembrolizumab alone. Analysis of tumor biopsies revealed increased CD8+ T-cell infiltration in the treated cohort, and the combination displayed a favorable safety and tolerability profile [187]. The clinical validation of NOX-mediated signaling as an actionable target in advanced solid tumors opens new perspectives into how remodeling the fibrotic and senescent microenvironment can enhance anti-tumor immunity.

Other nonspecific oncological clinical trials demonstrated the efficacy of a combined therapy to improve the ROS balance in primary biliary cholangitis and metabolic disease [188,189].

### 5.3. CCL2/CCR2 Signaling Axis

Given the pivotal role of the CCL2/CCR2 signaling axis in driving inflammatory monocyte trafficking, numerous CCR2 antagonists have been developed as potential therapeutics across a broad spectrum of inflammatory, metabolic, and cardiovascular disorders, including rheumatoid arthritis, asthma, diabetes, inflammatory bowel disease, and multiple sclerosis [190]. A recent clinical trial evaluated perioperative immunotherapeutic combinations aiming to target TME immunosuppressive mechanisms, specifically myeloid cell recruitment via CCR2/CCR5 pathways or IL-8 signaling, in patients with surgical candidates for early-stage/resectable solid tumors. No results of this study have been published [191].

Collectively, these emerging clinical findings demonstrate that targeting underlying mechanisms of cellular stress represents a feasible, safe, and viable paradigm for mitigating biological dysfunction, hypercoagulability, and therapy-associated toxicities in aging and oncology populations. However, further studies in larger patient cohorts are required to definitively establish their therapeutic efficacy and clinical benefit (Table 2).

## 6. Conclusions

**Figure 5:**
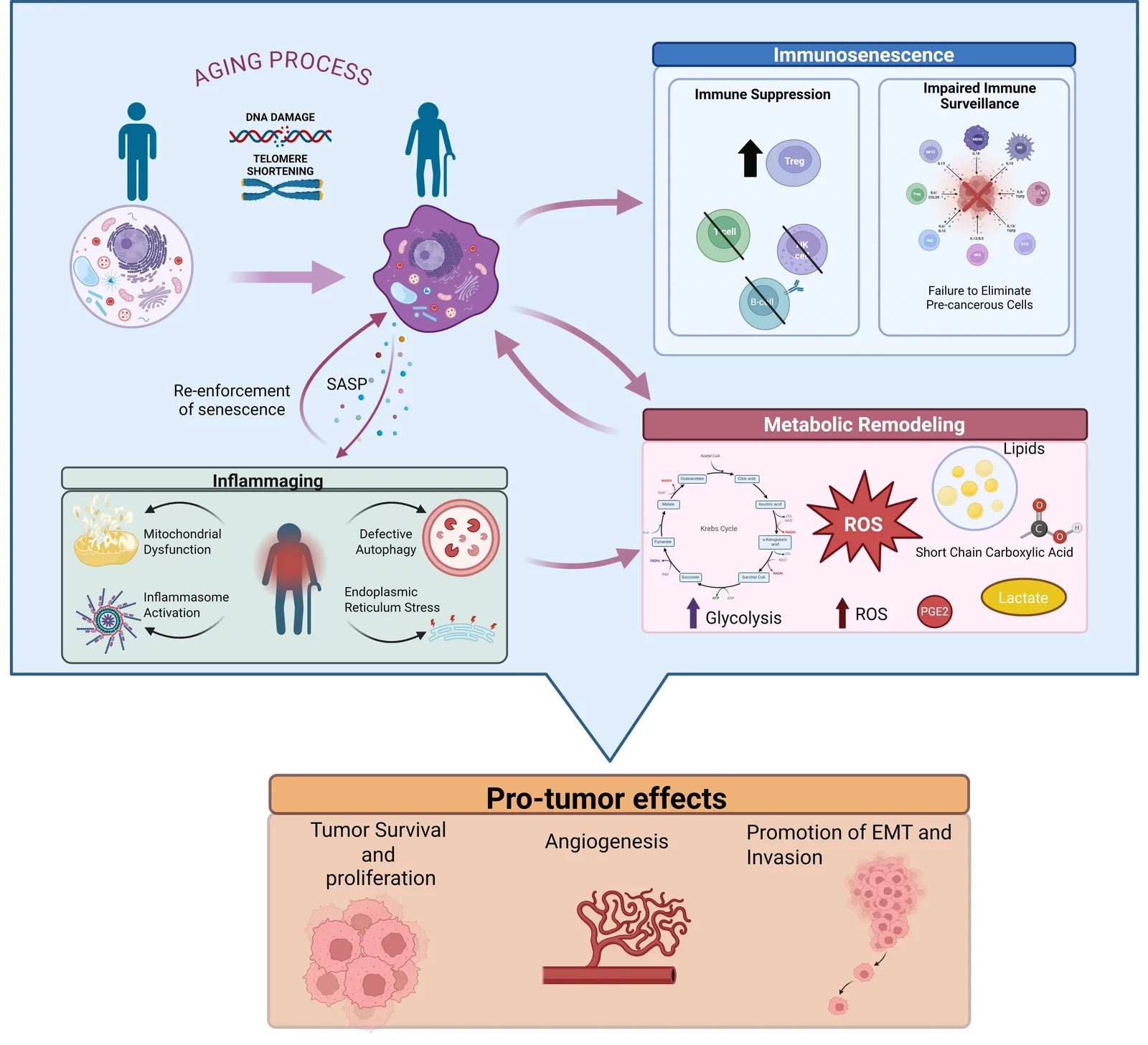
**Crosstalk between cellular senescence, immunosenescence, metabolic remodeling, and tumor progression.** Driven by the organismal aging process, intrinsic stress signals such as persistent DNA damage and telomere shortening induce cellular senescence. Senescent cells release SASP, which autocrinally reinforces the cell cycle arrest while paracrinally fueling chronic, systemic low-grade inflammation (Inflammaging). This inflammatory niche is supported by organelle dysfunction, including mitochondrial decay, defective autophagy, endoplasmic reticulum (ER) stress, and inflammasome activation. Concurrently, aging impairs the broader immune system through immunosenescence. This systemic immune suppression is marked by an expansion of immunosuppressive regulatory T cells (Treg) and blunted cytotoxic activity in effector T cells and Natural Killer (NK) cells. Together, these defects dismantle effective immune surveillance, compromising the host’s ability to recognize and eliminate pre-cancerous senescent cells before they cause further damage. Driven by chronic inflammaging, the microenvironment undergoes extensive metabolic remodeling. Cellular energy production shifts toward enhanced glycolysis and altered metabolic flux. Beyond classical protein signaling, senescent cells discharge a “Metabolic SASP”, a cocktail of non-protein small-molecule mediators such as extracellular lactate, short-chain carboxylic acids, and bioactive lipids, that conditions the surrounding tissue. Ultimately, these interconnected cascades—senescence, inflammaging, immune escape, and metabolic rewiring—converge to establish a highly permissive, pro-tumorigenic niche. This altered environment actively promotes cancer progression, enhancing tumor cell survival and proliferation, stimulating neo-angiogenesis, and promoting EMT to facilitate local tissue invasion and metastasis. [Created in BioRender. Tommasi, S. (2026) https://BioRender.com/lovqc1a].

From a holistic perspective, persistent senescent cells transform the microenvironment to drive tumor progression. The accumulation of senescent cells stems from aging and chronic stress, such as persistent DNA damage or oncogene activation, which permanently arrests cell proliferation. When physiological immune clearance mechanisms fail, these cells persist as continuous microenvironmental sources of inflammation and dysfunction. Accumulated senescent cells establish a persistent SASP consisting of inflammatory cytokines (TNF-α, IL-6, IL-1β), growth factors, and MMPs. These SASP factors reshape the local immune landscape, converting immune-competent sites into immunosuppressive niches. An immunosuppressive TME limits the infiltration and activation of cytotoxic T cells and natural killer cells while expanding regulatory populations, such as regulatory T cells (Treg) and M2 macrophages. Reciprocal crosstalk among senescent cells, cancer cells, and stromal compartments drives metabolic adaptations, including enhanced aerobic glycolysis and altered lipid dynamics. This metabolic state fuels a self-sustaining feedback loop that reinforces SASP secretion. Under this chronic inflammatory, metabolically altered, and immunosuppressive selective pressure, precancerous lesions accumulate genetic alterations, increase clonal heterogeneity, and evolve into advanced, treatment-resistant malignancies (Figure 5).

The transition from physiological senescence to malignancy is not a sudden event but a gradual remodeling of the biological landscape, orchestrated by the persistent state of inflammaging. Specific biological signatures such as SASP, oxidative stress, and dysregulated biomarkers bridge the gap between chronological aging and cancer progression. Senescent cells do not remain silent. They actively dismantle tissue architecture through the chronic release of pro-inflammatory cytokines into a permissive, pro-tumorigenic “soil”. The exploration of chemokines like CXCL10 reveals the complexity of the TME. While these molecules can recruit anti-tumor T cells, their dysregulation in the aging context often shifts toward promoting metastasis and immune evasion, highlighting the need for age-specific therapeutic strategies. The age-associated decline in autophagy leads to a buildup of cellular waste, which triggers the NLRP3 inflammasome. When coupled with the overproduction of ROS from dysfunctional mitochondria and NOX enzymes, the resulting oxidative stress promotes DNA damage and suppresses anti-tumor immune responses. Ultimately, the inflammaging signature provides a mechanistic roadmap for understanding why cancer is fundamentally a disease of aging. Recognizing these circulating biomarkers, not just as indicators of decay, but as active participants in oncogenesis, opens new avenues for geroncology. The SASP score, a molecular “biological thermometer” developed by processing vast health databases using an advanced deep learning architecture, could serve as a valuable tool for assessing senescence trajectories and predicting organ-related comorbidities associated with cancer. Future interventions must move beyond targeting the tumor alone, focusing instead on “quenching” the systemic inflammatory fire and restoring the structural integrity of the aging microenvironment to prevent the transition from senescence to malignancy.

## Figures and Tables

**Table 1:** Categorization and biological roles of key senescence-associated secretory phenotype (SASP) components.

SASP Categories	Components	Primary Activity	Pathological Activity
**Pro-Inflammatory Cytokines**	**IL-1α and IL-1β**	Promotion of chronic low-grade inflammation and reinforce senescence	Promotion of tumor growth, invasiveness, and therapeutic resistance by driving chronic inflammation within the tumor microenvironment
	**IL-6**	Pro-inflammatory mediation of the innate immune response and regulator of senescence	Mediation of Acute-phase cascade, metabolic homeostasis, chronic inflammatory disorders and cancer
	**TNF-α**	Mediation of local inflammation and support for senescence	Driver of malignant progression, tumor survival, and metastasis in the tumor microenvironment
**Matrix Metalloproteinases (MMPs)**	**MMP-3**	Degradation of extracellular matrix (ECM) components, disrupting tissue architecture and facilitating cell migration/invasion	Implication in tumor initiation, progression, and metastatic dissemination MMP-3 involvement in immune dysregulation and senescence
**Chemokines**	**CCL2 (MCP-1)**	Modulation of the recruitment and trafficking of multiple immune cell types to the local microenvironment and involvement into pathophysiology of aging	Driver of age-associated chronic diseases, tumor cell proliferation and chemoresistance.
	**CXCL10**	Regulation of chemotaxis induction of apoptosis, regulation of cell proliferation, and mediation of angiostatic activity and impact on the aging immune system	Pleiotropic regulator of cellular growth dynamics
**Metabolic mediators**	**Lactate**	ROS production, histone lactylation	Sustain the senescent state
	**Lipid mediators**	Products of COX2 activity	Sustaining a chronic state of inflammaging. Progressive accumulation of aged neutrophils

**Table 2:** Overview of interventional clinical trials.

NCT Number	Study Status	Pathology	Phases	Enrollment	Study Type
NCT00863655	COMPLETED	Breast Cancer	PHASE3	724	INTERVENTIONAL
NCT01062399	COMPLETED	Brain and Central Nervous System Tumors	PHASE1|PHASE2	279	INTERVENTIONAL
NCT02195232	COMPLETED	Thromboembolism of Vein VTE in Colorectal Cancer|Thromboembolism of Vein in Pancreatic Cancer|Thromboembolism of Vein in Non-small Cell Lung Cancer	PHASE2|PHASE3	64	INTERVENTIONAL
NCT02874989	COMPLETED	Idiopathic Pulmonary Fibrosis (IPF)	PHASE1	26	INTERVENTIONAL
NCT02900716	COMPLETED	Chronic Lymphocytic Leukemia|B-Cell Lymphoma	PHASE1	48	INTERVENTIONAL
NCT04123379	COMPLETED	Non-small Cell Lung Cancer|Hepatocellular Carcinoma	PHASE2	48	INTERVENTIONAL
NCT05014672	COMPLETED	Primary Biliary Cholangitis|Liver Stiffness	PHASE2	76	INTERVENTIONAL
NCT05323656	COMPLETED	Squamous Cell Carcinoma of Head and Neck	PHASE2	55	INTERVENTIONAL
NCT06940297	RECRUITING	Recurrent Multiple Myeloma|Refractory Multiple Myeloma	PHASE2	44	INTERVENTIONAL
NCT07619950	NOT_YET_RECRUITING	Neoplasms of Bone and Articular Cartilage With Unspecified Anatomical Site	PHASE2	94	INTERVENTIONAL

## Data Availability

No new data were created or analyzed in this study. Data sharing is not applicable to this article.

## Abbreviations

The following abbreviations are used in this manuscript:

ALS	Amyotrophic Lateral Sclerosis
BAG	Brain Age Gap
BC	Breast Cancer
bFGF	Basic fibroblast growth factor
CCL2	Chemokine ligand
CCR2	CCL2 receptor
CK17	Cytokeratin 17
CMML	Chronic myelomonocytic leukemia
COX2	Cyclooxygenase-2
CRC	Colorectal Cancer
CXCL10	Chemokine interferon-γ–inducible protein of 10 kDa
CXCR3	C-X-C motif chemokine receptor 3
DAMPs	Damage-associated molecular patterns
ECM	Extracellular matrix
EMT	Epithelial–mesenchymal transition
FADD	Fas-associated death domain
GAET	Guided Autoencoders with Transformers
HCC	Hepatocellular carcinoma
HIF-1α	Hypoxia-Inducible Factor 1-alpha
HPV	High Risk human papillomavirus
IFN-γ	Interferon gamma
IL	Interleukins
IL-18Rα	IL-18 receptor α
IL-18Rβ	IL-18 receptor β
IL-1R	IL-1 receptor
IL-1Rα	Inhibitor IL-1 receptor antagonist
IPF	Idiopathic pulmonary fibrosis
LE8	Life’s Essential 8
MCP-1	Monocyte chemoattractant protein-1
MDR	Multi-drug-resistant
MDR1	Multi-Drug Resistance 1
MDSCs	Myeloid-Derived Suppressor Cells
MMP-3	Matrix metalloproteinase-3
MMPs	Matrix metalloproteinases
MRP	Multidrug resistance-associated protein
mtDNA	mitochondrial DNA
mTOR	Mechanistic target of rapamycin
NADPH	Nicotinamide Adenine Dinucleotide Phosphate
NDRG1	N-Myc Downstream Regulated 1
NF-κB	Nuclear factor kappa-light-chain-enhancer of activated B cells
NK	Natural killer
NLRP3	NOD-, LRR- and pyrin domain-containing protein 3
NO	Nitric Oxide
NOX	Nicotinamide Adenine Dinucleotide Phosphate (NADPH) oxidases
NRF2	Nuclear factor erythroid 2-related factor 2
OS	Overall Survival
PAMPs	Pathogen-associated molecular patterns
PCNA	Proliferating cell nuclear antigen
PDK4	Pyruvate dehydrogenase kinase 4
PGE2	Prostaglandin E2
ROS	Reactive oxygen species
SASP	Senescence-associated secretory phenotype
TGFBR1	Transforming Growth Factor-beta receptor 1
TGFβ	Transforming Growth Factor-beta
TIMPs	Tissue inhibitors of metalloproteinases
TME	Tumor Microenvironment
TNFRI	TNF receptor I
TNFRII	TNF receptor II
TNF-α	Tumor Necrosis Factor-alpha
TRADD	TNF receptor-associated death domain
UKB-PPP	UK Biobank Pharma Proteomics Project

## References

[1] Wyss-Coray T., Topol E.J. (2026). Biological Aging Clocks in Health and Disease. Nat. Med..

[2] Miao H., Liu S., Wang Z., Ke Y., Cheng L., Yu W., Yu D., Zhang K., Gao Y., Sun Z. (2025). Artificial Intelligence-Derived Retinal Age Gap as a Marker for Reproductive Aging in Women. npj Digit. Med..

[3] Li D., Ju F., Wang H., Fan C., Jacob J.C., Gul S., Zaliani A., Wartmann T., Polidori M.C., Bruns C.J. (2023). Combination of the Biomarkers for Aging and Cancer?—Challenges and Current Status. Transl. Oncol..

[4] Mishra P., Banerjee D., Ben-Baruch A. (2010). Chemokines at the Crossroads of Tumor-Fibroblast Interactions That Promote Malignancy. J. Leukoc. Biol..

[5] Bafei S.E.C., Shen C. (2023). Biomarkers Selection and Mathematical Modeling in Biological Age Estimation. NPJ Aging.

[6] Zhang R., Yi F., Mao H., Huang Z., Wang K., Zhang J. (2025). Brain Age Gap as a Predictive Biomarker That Links Aging, Lifestyle, and Neuropsychiatric Health. Commun. Med..

[7] Sedrak M.S., Cohen H.J. (2023). The Aging–Cancer Cycle: Mechanisms and Opportunities for Intervention. J. Gerontol. A Biol. Sci. Med. Sci..

[8] Levine M.E., Lu A.T., Quach A., Chen B.H., Assimes T.L., Bandinelli S., Hou L., Baccarelli A.A., Stewart J.D., Li Y. (2018). An Epigenetic Biomarker of Aging for Lifespan and Healthspan. Aging.

[9] Bian L., Ma Z., Fu X., Ji C., Wang T., Yan C., Dai J., Ma H., Hu Z., Shen H. (2024). Associations of Combined Phenotypic Aging and Genetic Risk with Incident Cancer: A Prospective Cohort Study. eLife.

[10] Tian R., Zong X., Ren D., Tica S., Hong D., Oduyale O., Buenrostro J.D., Govindan R., Cao Y. (2026). Biological Aging and Generational Shifts in Early-Onset Cancer Risk. Nat. Med..

[11] Angarola B.L., Sharma S., Katiyar N., Kang H.G., Nehar-Belaid D., Park S., Gott R., Eryilmaz G.N., LaBarge M.A., Palucka K. (2024). Comprehensive Single-Cell Aging Atlas of Healthy Mammary Tissues Reveals Shared Epigenomic and Transcriptomic Signatures of Aging and Cancer. Nat. Aging.

[12] Campisi J., d’Adda Di Fagagna F. (2007). Cellular Senescence: When Bad Things Happen to Good Cells. Nat. Rev. Mol. Cell Biol..

[13] Lam Y., Gu J., Yin P. (2025). Cellular Senescence in Cancer: Unveiling Dual Roles, Tumor Microenvironment Dynamics and Therapeutic Innovations (Review). Oncol. Lett..

[14] Liu L., Huang H., Cheng B., Xie H., Peng W., Cui H., Liang J., Cao M., Yang Y., Chen W. (2025). Revealing the Role of Cancer-Associated Fibroblast Senescence in Prognosis and Immune Landscape in Pancreatic Cancer. iScience.

[15] Omer A., Di Marco S., Gallouzi I.-E. (2021). The Senescence-Associated Secretory Phenotype as a Driver of Tumor Growth: Does G3BP1 Hold the Key?. Mol. Cell. Oncol..

[16] Yoshimoto S., Loo T.M., Atarashi K., Kanda H., Sato S., Oyadomari S., Iwakura Y., Oshima K., Morita H., Hattori M. (2013). Obesity-Induced Gut Microbial Metabolite Promotes Liver Cancer through Senescence Secretome. Nature.

[17] Acosta J.C., Banito A., Wuestefeld T., Georgilis A., Janich P., Morton J.P., Athineos D., Kang T.-W., Lasitschka F., Andrulis M. (2013). A Complex Secretory Program Orchestrated by the Inflammasome Controls Paracrine Senescence. Nat. Cell Biol..

[18] Kuilman T., Michaloglou C., Vredeveld L.C.W., Douma S., Van Doorn R., Desmet C.J., Aarden L.A., Mooi W.J., Peeper D.S. (2008). Oncogene-Induced Senescence Relayed by an Interleukin-Dependent Inflammatory Network. Cell.

[19] González-Gualda E., Reinius M.A.V., Macias D., Morsli S., Ge J., Olan I., Martín J.E., Ou H.-L., Hartono M., Puerto-Camacho M.P. (2026). Treatment Resistance to Platinum-Based Chemotherapy in Lung and Ovarian Cancer Is Driven by a Targetable TGFβ Senescent Secretome. Nat. Aging.

[20] Hernandez-Segura A., Nehme J., Demaria M. (2018). Hallmarks of Cellular Senescence. Trends Cell Biol..

[21] Kumari R., Jat P. (2021). Mechanisms of Cellular Senescence: Cell Cycle Arrest and Senescence Associated Secretory Phenotype. Front. Cell Dev. Biol..

[22] Grill S., Nandakumar J. (2021). Molecular Mechanisms of Telomere Biology Disorders. J. Biol. Chem..

[23] d’Adda Di Fagagna F. (2008). Living on a Break: Cellular Senescence as a DNA-Damage Response. Nat. Rev. Cancer.

[24] Navarro C., Salazar J., Díaz M.P., Chacin M., Santeliz R., Vera I., D′Marco L., Parra H., Bernal M.C., Castro A. (2023). Intrinsic and Environmental Basis of Aging: A Narrative Review. Heliyon.

[25] Pawelec G. (2018). Age and Immunity: What Is “Immunosenescence”?. Exp. Gerontol..

[26] Su J., Jin H. (2026). Advances in Skin Aging: Integrating Epigenetic, Cellular, and Immune Mechanisms for Targeted Therapy. Immun. Ageing.

[27] Teissier T., Boulanger E., Cox L.S. (2022). Interconnections between Inflammageing and Immunosenescence during Ageing. Cells.

[28] Nguyen T.Q.T., Cho K.A. (2025). Targeting Immunosenescence and Inflammaging: Advancing Longevity Research. Exp. Mol. Med..

[29] Liu Z., Liang Q., Ren Y., Guo C., Ge X., Wang L., Cheng Q., Luo P., Zhang Y., Han X. (2023). Immunosenescence: Molecular Mechanisms and Diseases. Signal Transduct. Target. Ther..

[30] Ansari W.A., Srivastava K., Nasibullah M., Khan M.F. (2025). Reactive Oxygen Species (ROS): Sources, Generation, Disease Pathophysiology, and Antioxidants. Discov. Chem..

[31] Rea I.M., Gibson D.S., McGilligan V., McNerlan S.E., Alexander H.D., Ross O.A. (2018). Age and Age-Related Diseases: Role of Inflammation Triggers and Cytokines. Front. Immunol..

[32] Takac I., Schröder K., Brandes R.P. (2012). The Nox Family of NADPH Oxidases: Friend or Foe of the Vascular System?. Curr. Hypertens. Rep..

[33] Pérez De La Lastra J.M., Andrés C.M.C., Bustamante Munguira E., Juan C.A., Pérez-Lebeña E. (2026). Beyond Antioxidants: How ROS-RNS-RSS Microdomains Encode Redox Information in Health and Disease. Free Radic. Biol. Med..

[34] Reis J., Gorgulla C., Massari M., Marchese S., Valente S., Noce B., Basile L., Törner R., Cox H., Viennet T. (2023). Targeting ROS Production through Inhibition of NADPH Oxidases. Nat. Chem. Biol..

[35] Lei H., Zhao T., Zhang J., Long Y., Chen Y., Zhu Y., Meng Y., Tang Y., Liu X., Sun J. (2025). NRF1-Mediated Innate Immune Response Drives Inflammaging. Nat. Commun..

[36] Wilson W.R., Hay M.P. (2011). Targeting Hypoxia in Cancer Therapy. Nat. Rev. Cancer.

[37] Chen Y., Hou J., Tian M., Hao E., Zhang Y., Huang D., Zhang T., Ma Y. (2026). Time-Dependent Effects of Hypoxia on Mitochondrial Dynamics, Dysfunction and Apoptosis in C8-D1A Astrocytes. Open Life Sci..

[38] Franchi L., Muñoz-Planillo R., Núñez G. (2012). Sensing and Reacting to Microbes through the Inflammasomes. Nat. Immunol..

[39] Cruz C.M., Rinna A., Forman H.J., Ventura A.L.M., Persechini P.M., Ojcius D.M. (2007). ATP Activates a Reactive Oxygen Species-Dependent Oxidative Stress Response and Secretion of Proinflammatory Cytokines in Macrophages. J. Biol. Chem..

[40] Schroder K., Tschopp J. (2010). The Inflammasomes. Cell.

[41] Zubkova E., Kalinin A., Bolotskaya A., Beloglazova I., Menshikov M. (2024). Autophagy-Dependent Secretion: Crosstalk between Autophagy and Exosome Biogenesis. Curr. Issues Mol. Biol..

[42] Hansen M. (2012). Autophagy—An Emerging Anti-Aging Mechanism?. J. Clin. Exp. Pathol..

[43] Cuervo A.M., Macian F. (2014). Autophagy and the Immune Function in Aging. Curr. Opin. Immunol..

[44] Zhang Z., Zhao Z., Xie S., Guo B., Cai C., Wu C., Hao X., Ni J., Cui H., Xiong M. (2026). NLRC4 and NLRP3 in Tissues as Potential Biomarkers for the Diagnosis of Non-Small Cell Lung Cancer in Pulmonary Tuberculosis. Front. Cell. Infect. Microbiol..

[45] Lu K., Cui Z., Fei X., Zhang J., Fang S., Wang Y., Chen Y., Wu Y., Xia D. (2026). Low-Dose PFNA Exposure Exacerbates Intestinal Inflammation via Caspase-11-Dependent Noncanonical NLRP3 Inflammasome Activation in Macrophages. J. Adv. Res..

[46] Daley D., Mani V.R., Mohan N., Akkad N., Pandian G.S.D.B., Savadkar S., Lee K.B., Torres-Hernandez A., Aykut B., Diskin B. (2017). NLRP3 Signaling Drives Macrophage-Induced Adaptive Immune Suppression in Pancreatic Carcinoma. J. Exp. Med..

[47] Hurtado-Navarro L., Cuenca-Zamora E.J., Zamora L., Bellosillo B., Such E., Soler-Espejo E., Martínez-Banaclocha H., Hernández-Rivas J.M., Marco-Ayala J., Martínez-Alarcón L. (2023). NLRP3 Inflammasome Activation and Symptom Burden in KRAS-Mutated CMML Patients Is Reverted by IL-1 Blocking Therapy. Cell Rep. Med..

[48] Allen I.C., TeKippe E.M., Woodford R.-M.T., Uronis J.M., Holl E.K., Rogers A.B., Herfarth H.H., Jobin C., Ting J.P.-Y. (2010). The NLRP3 Inflammasome Functions as a Negative Regulator of Tumorigenesis during Colitis-Associated Cancer. J. Exp. Med..

[49] Lu F., Zhao Y., Pang Y., Ji M., Sun Y., Wang H., Zou J., Wang Y., Li G., Sun T. (2021). NLRP3 Inflammasome Upregulates PD-L1 Expression and Contributes to Immune Suppression in Lymphoma. Cancer Lett..

[50] Abdulkhaleq L.A., Assi M.A., Abdullah R., Zamri-Saad M., Taufiq-Yap Y.H., Hezmee M.N.M. (2018). The Crucial Roles of Inflammatory Mediators in Inflammation: A Review. Vet. World.

[51] King T.C. (2007). Inflammation, Inflammatory Mediators, and Immune-Mediated Disease. Elsevier’s Integrated Pathology.

[52] Sims J.E., Smith D.E. (2010). The IL-1 Family: Regulators of Immunity. Nat. Rev. Immunol..

[53] Cavallone L. (2003). The Role of IL-1 Gene Cluster in Longevity: A Study in Italian Population. Mech. Ageing Dev..

[54] Jylhä M., Paavilainen P., Lehtimäki T., Goebeler S., Karhunen P.J., Hervonen A., Hurme M. (2007). Interleukin-1 Receptor Antagonist, Interleukin-6, and C-Reactive Protein as Predictors of Mortality in Nonagenarians: The Vitality 90+ Study. J. Gerontol. A Biol. Sci. Med. Sci..

[55] Smith D.E. (2010). The Biological Paths of IL-1 Family Members IL-18 and IL-33. J. Leukoc. Biol..

[56] Dinarello C.A., Novick D., Kim S., Kaplanski G. (2013). Interleukin-18 and IL-18 Binding Protein. Front. Immunol..

[57] Salvioli S., Basile M.S., Bencivenga L., Carrino S., Conte M., Damanti S., De Lorenzo R., Fiorenzato E., Gialluisi A., Ingannato A. (2023). Biomarkers of Aging in Frailty and Age-Associated Disorders: State of the Art and Future Perspective. Ageing Res. Rev..

[58] Skoie I.M., Dalen I., Kvivik I., Bårdsen K., Omdal R. (2020). Fatigue in Patients with Plaque-Type Psoriasis: Lack of an Association with Plasma Cytokines. Eur. J. Dermatol..

[59] Wu H., Zhang Z., Ma Y., Chen F., Xiong P., Xie Z., Ding G., Yu J., Wang K. (2022). Dynamic Immune and Exosome Transcriptomic Responses in Patients Undergoing Psychostimulant Methamphetamine Withdrawal. Front. Cell. Neurosci..

[60] Herder C., Brunner E.J., Rathmann W., Strassburger K., Tabák A.G., Schloot N.C., Witte D.R. (2009). Elevated Levels of the Anti-Inflammatory Interleukin-1 Receptor Antagonist Precede the Onset of Type 2 Diabetes. Diabetes Care.

[61] Bruni D., Angell H.K., Galon J. (2020). The Immune Contexture and Immunoscore in Cancer Prognosis and Therapeutic Efficacy. Nat. Rev. Cancer.

[62] Mantovani A., Dinarello C.A., Molgora M., Garlanda C. (2019). Interleukin-1 and Related Cytokines in the Regulation of Inflammation and Immunity. Immunity.

[63] Tu S., Bhagat G., Cui G., Takaishi S., Kurt-Jones E.A., Rickman B., Betz K.S., Penz-Oesterreicher M., Bjorkdahl O., Fox J.G. (2008). Overexpression of Interleukin-1β Induces Gastric Inflammation and Cancer and Mobilizes Myeloid-Derived Suppressor Cells in Mice. Cancer Cell.

[64] Aleman H., Esparza J., Ramirez F.A., Astiazaran H., Payette H. (2011). Longitudinal Evidence on the Association between Interleukin-6 and C-Reactive Protein with the Loss of Total Appendicular Skeletal Muscle in Free-Living Older Men and Women. Age Ageing.

[65] Prašnikar E., Borišek J., Perdih A. (2021). Senescent Cells as Promising Targets to Tackle Age-Related Diseases. Ageing Res. Rev..

[66] Rodier F., Coppé J.-P., Patil C.K., Hoeijmakers W.A.M., Muñoz D.P., Raza S.R., Freund A., Campeau E., Davalos A.R., Campisi J. (2009). Persistent DNA Damage Signalling Triggers Senescence-Associated Inflammatory Cytokine Secretion. Nat. Cell Biol..

[67] Hoare M., Das T., Alexander G. (2010). Ageing, Telomeres, Senescence, and Liver Injury. J. Hepatol..

[68] Herbstein F., Sapochnik M., Attorresi A., Pollak C., Senin S., Gonilski-Pacin D., Ciancio del Giudice N., Fiz M., Elguero B., Fuertes M. (2024). The SASP Factor IL-6 Sustains Cell-autonomous Senescent Cells via a cGAS-STING-NFκB Intracrine Senescent Noncanonical Pathway. Aging Cell.

[69] Martins F., Martínez-Flores R., Pereira V., González-Arriagada W.A., Martins M.D., Hunter K.D., Niklander S.E., Bologna-Molina R. (2026). Image Segmentation-Based Analysis Reveals Correlation between IL-1RA Overexpression and Worse Survival Outcomes in Oral Squamous Cell Carcinoma. Sci. Rep..

[70] Kawano Y., Kawano H., LaMere M.W., LaMere E.A., Byun D.K., McGrath K.E., Palis J., Bajaj J., Liesveld J.L., Katayama Y. (2025). IL-1R1 and IL-18 Signals Regulate Mesenchymal Stromal Cells in an Aged Murine Model of Myelodysplastic Syndromes. Blood.

[71] Sassun R., Roufael F., Crippa J., Magistro C., Bertoglio C., Achilli P., Dinuzzi V.P., Montroni I., Maggioni D., Spinelli A. (2025). Impact of Elective Surgery on Tumor Necrosis Factor-α, Interleukin-6 and Quality of Life in Uncomplicated Diverticular Disease. Colorectal Dis..

[72] Li X., Zhong J., Deng X., Guo X., Lu Y., Lin J., Huang X., Wang C. (2021). Targeting Myeloid-Derived Suppressor Cells to Enhance the Antitumor Efficacy of Immune Checkpoint Blockade Therapy. Front. Immunol..

[73] Ullah A., Singla R.K., Cao D., Chen B., Shen B. (2025). Age-Related and Postmenopausal Breast Cancer Progression and Treatment Management: The Significance of pro-Inflammatory Cytokines and CXC Chemokines. Genes Dis..

[74] Balkwill F., Charles K.A., Mantovani A. (2005). Smoldering and Polarized Inflammation in the Initiation and Promotion of Malignant Disease. Cancer Cell.

[75] Wang Y., Liang M., Mao Y., Zhu W., Shen X., Guan W. (2026). Aging Modulation of the Immune System and Immunotherapy Efficacy in Cancer. Front. Immunol..

[76] Ferrucci L., Corsi A., Lauretani F., Bandinelli S., Bartali B., Taub D.D., Guralnik J.M., Longo D.L. (2005). The Origins of Age-Related Proinflammatory State. Blood.

[77] Watts A.D., Hunt N.H., Madigan M.C., Chaudhri G. (1999). Soluble TNF-α Receptors Bind and Neutralize over-Expressed Transmembrane TNF-α on Macrophages, but Do Not Inhibit Its Processing. J. Leukoc. Biol..

[78] Nadir Y., Brenner B. (2018). Cancer and Thrombosis—New Insights. Rambam Maimonides Med. J..

[79] Roberts N.J., Zhou S., Diaz L.A., Holdhoff M. (2011). Systemic Use of Tumor Necrosis Factor Alpha as an Anticancer Agent. Oncotarget.

[80] Li Y., Ye R., Dai H., Lin J., Cheng Y., Zhou Y., Lu Y. (2025). Exploring TNFR1: From Discovery to Targeted Therapy Development. J. Transl. Med..

[81] Wajant H., Siegmund D. (2019). TNFR1 and TNFR2 in the Control of the Life and Death Balance of Macrophages. Front. Cell Dev. Biol..

[82] Yu K., Yu C., Jiao L., Miao K., Ni L., Rao X., Zhou L., Zhao C. (2022). The Function and Therapeutic Implications of TNF Signaling in MDSCs. Biomolecules.

[83] Kumari N., Reabroi S., North B.J. (2021). Unraveling the Molecular Nexus between GPCRs, ERS, and EMT. Mediat. Inflamm..

[84] Li H., Wang D., Singh L.S., Berk M., Tan H., Zhao Z., Steinmetz R., Kirmani K., Wei G., Xu Y. (2009). Abnormalities in Osteoclastogenesis and Decreased Tumorigenesis in Mice Deficient for Ovarian Cancer G Protein-Coupled Receptor 1. PLoS ONE.

[85] Tundealao S., Irwin M.R., Cole S., Blair C.K., Lu S.-E., Saraiya B., Kim I.Y., Guest D.D., Lin Y., An J. (2026). Biological Correlates of Cancer-Related Fatigue in Older Male Cancer Survivors. Transl. Psychiatry.

[86] Turner N., Mughal R., Warburton P., Oregan D., Ball S., Porter K. (2007). Mechanism of TNFα-Induced IL-1α, IL-1β and IL-6 Expression in Human Cardiac Fibroblasts: Effects of Statins and Thiazolidinediones. Cardiovasc. Res..

[87] Ruiz J.I., Lei X., Giordano S.H., Zhao H., Rajan S.S., Lin H., Suarez-Almazor M.E. (2025). Survival in Patients with Rheumatoid Arthritis and Recently Diagnosed Early-Stage Colorectal, Lung, or Prostate Cancer Receiving Tumour Necrosis Factor Inhibitors: A Retrospective Cohort Study. Lancet Rheumatol..

[88] Mozooni Z., Ghadyani R., Soleimani S., Ahangar E.R., Sheikhpour M., Haghighi M., Motallebi M., Movafagh A., Aghaei-Zarch S.M. (2024). TNF-α, and TNFRs in Gastrointestinal Cancers. Pathol. Res. Pract..

[89] Shen W.H., Zhou J.H., Broussard S.R., Freund G.G., Dantzer R., Kelley K.W. (2002). Proinflammatory Cytokines Block Growth of Breast Cancer Cells by Impairing Signals from a Growth Factor Receptor. Cancer Res..

[90] Haranaka K., Satomi N., Sakurai A. (1984). Antitumor Activity of Murine Tumor Necrosis Factor (TNF) against Transplanted Murine Tumors and Heterotransplanted Human Tumors in Nude Mice. Int. J. Cancer.

[91] Jones A.L., O’Brien M.E.R., Lorentzos A., Viner C., Hanrahan A., Moore J., Millar J.L., Gore M.E. (1992). A Randomised Phase II Study of Carmustine Alone or in Combination with Tomour Necrosis Factor in Patients with Advanced Melanoma. Cancer Chemother. Pharmacol..

[92] Chapman P.B., Lester T.J., Casper E.S., Gabrilove J.L., Wong G.Y., Kempin S.J., Gold P.J., Welt S., Warren R.S., Starnes H.F. (1987). Clinical Pharmacology of Recombinant Human Tumor Necrosis Factor in Patients with Advanced Cancer. J. Clin. Oncol..

[93] Enderlin M., Kleinmann E.V., Struyf S., Buracchi C., Vecchi A., Kinscherf R., Kiessling F., Paschek S., Sozzani S., Rommelaere J. (2009). TNF-α and the IFN-γ-Inducible Protein 10 (IP-10/CXCL-10) Delivered by Parvoviral Vectors Act in Synergy to Induce Antitumor Effects in Mouse Glioblastoma. Cancer Gene Ther..

[94] Yang L., Chen P., Luo S., Li J., Liu K., Hu H., Wei Y. (2009). CXC-chemokine-ligand-10 Gene Therapy Efficiently Inhibits the Growth of Cervical Carcinoma on the Basis of Its Anti-angiogenic and Antiviral Activity. Biotechnol. Appl. Biochem..

[95] Lo B.K.K., Yu M., Zloty D., Cowan B., Shapiro J., McElwee K.J. (2010). CXCR3/Ligands Are Significantly Involved in the Tumorigenesis of Basal Cell Carcinomas. Am. J. Pathol..

[96] Buccisano F., Maurillo L., Gattei V., Del Poeta G., Del Principe M.I., Fraboni F., Panetta P., Consalvo M.I., Campagna S., Giannì L. (2006). Optimal Post-Remission Therapy for Flow-Cytometry Minimal Residual Disease Positive Patients with Acute Myeloid Leukemia. Blood.

[97] Angiolillo A.L., Sgadari C., Taub D.D., Liao F., Farber J.M., Maheshwari S., Kleinman H.K., Reaman G.H., Tosato G. (1995). Human Interferon-Inducible Protein 10 Is a Potent Inhibitor of Angiogenesis in Vivo. J. Exp. Med..

[98] Ozga A.J., Chow M.T., Lopes M.E., Servis R.L., Di Pilato M., Dehio P., Lian J., Mempel T.R., Luster A.D. (2022). CXCL10 Chemokine Regulates Heterogeneity of the CD8+ T Cell Response and Viral Set Point during Chronic Infection. Immunity.

[99] Korsunsky I., Wei K., Pohin M., Kim E.Y., Barone F., Major T., Taylor E., Ravindran R., Kemble S., Watts G.F.M. (2022). Cross-Tissue, Single-Cell Stromal Atlas Identifies Shared Pathological Fibroblast Phenotypes in Four Chronic Inflammatory Diseases. Med.

[100] Hu X., Ni S., Zhao K., Qian J., Duan Y. (2022). Bioinformatics-Led Discovery of Osteoarthritis Biomarkers and Inflammatory Infiltrates. Front. Immunol..

[101] Zhang Q., Shan Y., Shen L., Ni Q., Wang D., Wen X., Xu H., Liu X., Zeng Z., Yang J. (2024). Renal Remodeling by CXCL10-CXCR3 Axis-Recruited Mesenchymal Stem Cells and Subsequent IL4I1 Secretion in Lupus Nephritis. Signal Transduct. Target. Ther..

[102] Li W., Wei H., Liu J., Zhao Z., Wang F., Qiao L., Yin S., Zhang C., Huo M. (2025). Exosomal Biglycan Promotes Gastric Cancer Progression via M2 Polarization and CXCL10-Mediated JAK/STAT1 Activation. Cancer Lett..

[103] Chai X., Chen J., Zhao Y., Chu W., Zhou J., Zhao Y., Hu Z., Zhu J., Zhu Y., Xu Z. (2026). G9a Epigenetically Suppresses CXCL10 Expression and Inhibits Anti-tumor Immunity in Hepatocellular Carcinoma. Int. J. Cancer.

[104] House I.G., Savas P., Lai J., Chen A.X.Y., Oliver A.J., Teo Z.L., Todd K.L., Henderson M.A., Giuffrida L., Petley E.V. (2020). Macrophage-Derived CXCL9 and CXCL10 Are Required for Antitumor Immune Responses Following Immune Checkpoint Blockade. Clin. Cancer Res..

[105] Zhang B., Yuan X., Meng L., Liu Y., Wang Y., Chen Z., Zeng H., Zhang X., Peng Z., Zhang H. (2026). Tumor-Derived CXCL10 and CCL20 Polarize the Immune Microenvironment in Nasopharyngeal Carcinoma via Competitive Recruitment of Effector T Cells and Tregs. Int. J. Med. Sci..

[106] Shinn C.K., Saddawi-Konefka R., Salanga C.L., Schokrpur S., Gutkind J.S., Handel T.M. (2026). Exploiting the CXCR3/CXCL10 Axis Overrides Tumor Immune Suppression by Enhancing Immune Trafficking and Effector Cell Priming in HNSCC. Proc. Natl. Acad. Sci. USA.

[107] Sato E., Fujimoto J., Tamaya T. (2007). Expression of Interferon-Gamma-Inducible Protein 10 Related to Angiogenesis in Uterine Endometrial Cancers. Oncology.

[108] Dai L., Fan G., Xie T., Li L., Tang L., Chen H., Shi Y., Han X. (2024). Single-Cell and Spatial Transcriptomics Reveal a High Glycolysis B Cell and Tumor-Associated Macrophages Cluster Correlated with Poor Prognosis and Exhausted Immune Microenvironment in Diffuse Large B-Cell Lymphoma. Biomark. Res..

[109] Higuchi T., Hashida Y., Matsuo K., Kitahata K., Ujihara T., Murakami I., Nakayama T., Daibata M. (2023). EBV-positive Pyothorax-associated Lymphoma Expresses CXCL9 and CXCL10 Chemokines That Attract Cytotoxic Lymphocytes via CXCR3. Cancer Sci..

[110] Jung H., Paust S. (2024). Chemokines in the Tumor Microenvironment: Implications for Lung Cancer and Immunotherapy. Front. Immunol..

[111] Martina S., Martina V., Monika M., Jan P., Libor K., Ilja S. (2009). Angiostatic versus Angiogenic Chemokines in IPF and EAA. Respir. Med..

[112] Persano L., Crescenzi M., Indraccolo S. (2007). Anti-Angiogenic Gene Therapy of Cancer: Current Status and Future Prospects. Mol. Asp. Med..

[113] Fujita M., Zhu X., Ueda R., Sasaki K., Kohanbash G., Kastenhuber E.R., McDonald H.A., Gibson G.A., Watkins S.C., Muthuswamy R. (2009). Effective Immunotherapy against Murine Gliomas Using Type 1 Polarizing Dendritic Cells—Significant Roles of CXCL10. Cancer Res..

[114] Romagnani P., Lasagni L., Annunziato F., Serio M., Romagnani S. (2004). CXC Chemokines: The Regulatory Link between Inflammation and Angiogenesis. Trends Immunol..

[115] Datta D., Flaxenburg J.A., Laxmanan S., Geehan C., Grimm M., Waaga-Gasser A.M., Briscoe D.M., Pal S. (2006). Ras-Induced Modulation of CXCL10 and Its Receptor Splice Variant CXCR3-B in MDA-MB-435 and MCF-7 Cells: Relevance for the Development of Human Breast Cancer. Cancer Res..

[116] Song W., Yin H., Han C., Mao Q., Tang J., Ji Z., Yan X., Wang L., Liu S., Ai C. (2021). The Role of CXCL10 in Prognosis of Patients with Colon Cancer and Tumor Microenvironment Remodeling. Medicine.

[117] Madkhali O.A., Moni S.S., Almoshari Y., Sabei F.Y., Safhi A.Y. (2025). Dual Role of CXCL10 in Cancer Progression: Implications for Immunotherapy and Targeted Treatment. Cancer Biol. Ther..

[118] Liu J., Yin T., Wu Y., Peng X., Zhang J., Zhan X. (2026). The Dual Role of the CXCL9/10/11-CXCR3 Axis in the Tumor Immune Microenvironment: Mechanisms, Therapeutic Implications, and Clinical Translation. Pharmacol. Res..

[119] Vinet J., De Jong E.K., Boddeke H.W.G.M., Stanulovic V., Brouwer N., Granic I., Eisel U.L.M., Liem R.S.B., Biber K. (2010). Expression of CXCL10 in Cultured Cortical Neurons. J. Neurochem..

[120] Yin S., Yeung K.T., Mamde S., Lin K., Gandhi A., Lei X., Davis A., Arnold R., Yang J., Adams P.D. (2025). Aging Alters Tumor Cell—T Cell Crosstalk to Promote Breast Cancer Progression. bioRxiv.

[121] Del Prado-Montero J., Guevara-Martínez J., Cantero-Cid R., Mata-Martínez P., Cueto F.J., Lozano-Rodríguez R., Terrón-Arcos V., Abad-Moret R., Díaz-Serrano E., De Diego R.P. (2025). Age-Stratified Circulating Immune Signatures Reveal Non-Invasive Biomarkers for Colorectal Cancer Detection. Immun. Ageing.

[122] Yoshimura T., Li C., Wang Y., Matsukawa A. (2023). The Chemokine Monocyte Chemoattractant Protein-1/CCL2 Is a Promoter of Breast Cancer Metastasis. Cell. Mol. Immunol..

[123] Gschwandtner M., Derler R., Midwood K.S. (2019). More Than Just Attractive: How CCL2 Influences Myeloid Cell Behavior Beyond Chemotaxis. Front. Immunol..

[124] Huma Z.E., Sanchez J., Lim H.D., Bridgford J.L., Huang C., Parker B.J., Pazhamalil J.G., Porebski B.T., Pfleger K.D.G., Lane J.R. (2017). Key Determinants of Selective Binding and Activation by the Monocyte Chemoattractant Proteins at the Chemokine Receptor CCR2. Sci. Signal..

[125] Xu M., Wang Y., Xia R., Wei Y., Wei X. (2021). Role of the CCL2-CCR2 Signalling Axis in Cancer: Mechanisms and Therapeutic Targeting. Cell Prolif..

[126] Gok Yavuz B., Gunaydin G., Gedik M.E., Kosemehmetoglu K., Karakoc D., Ozgur F., Guc D. (2019). Cancer Associated Fibroblasts Sculpt Tumour Microenvironment by Recruiting Monocytes and Inducing Immunosuppressive PD-1+ TAMs. Sci. Rep..

[127] Guo D., Zhu W., Qiu H. (2024). C-C Motif Chemokine Ligand 2 and Chemokine Receptor 2 in Cardiovascular and Neural Aging and Aging-Related Diseases. Int. J. Mol. Sci..

[128] Loberg R.D., Day L.L., Harwood J., Ying C., St. John L.N., Giles R., Neeley C.K., Pienta K.J. (2006). CCL2 Is a Potent Regulator of Prostate Cancer Cell Migration and Proliferation. Neoplasia.

[129] Hughes C.E., Nibbs R.J.B. (2018). A Guide to Chemokines and Their Receptors. FEBS J..

[130] Goenka A., Khan F., Verma B., Sinha P., Dmello C.C., Jogalekar M.P., Gangadaran P., Ahn B. (2023). Tumor Microenvironment Signaling and Therapeutics in Cancer Progression. Cancer Commun..

[131] Hajibabaie F., Abedpoor N., Maleki B., Boshtam M., Shenavar N., Zarrabi A., Babakhani M., Sharifi M., Shariati L., Rahimmanesh I. (2026). Targeting the CCL2/CCR2 Axis in Colorectal Cancer: Immunopharmacological Perspectives and Therapeutic Strategies. Crit. Rev. Oncol. Hematol..

[132] Chen X., Yang M., Yin J., Li P., Zeng S., Zheng G., He Z., Liu H., Wang Q., Zhang F. (2022). Tumor-Associated Macrophages Promote Epithelial–Mesenchymal Transition and the Cancer Stem Cell Properties in Triple-Negative Breast Cancer through CCL2/AKT/β-Catenin Signaling. Cell Commun. Signal..

[133] Marsland M., Jiang C.C., Faulkner S., Steigler A., McEwan K., Jobling P., Oldmeadow C., Delahunt B., Denham J.W., Hondermarck H. (2024). CCL2/CCR2 Expression in Locally Advanced Prostate Cancer and Patient Long-Term Outcome: 10-Year Results from the TROG 03.04 RADAR Trial. Cancers.

[134] Drosslerova M., Sterclova M., Taskova A., Hytych V., Richterova E., Bruzova M., Spunda M., Komarc M., Koziar Vasakova M. (2023). CCL2, CCL8, CXCL12 Chemokines in Resectable Non-Small Cell Lung Cancer (NSCLC). Biomed. Pap. Med. Fac. Univ. Palacký Olomouc Czech. Repub..

[135] Xie M., Lin Z., Ji X., Luo X., Zhang Z., Sun M., Chen X., Zhang B., Liang H., Liu D. (2023). FGF19/FGFR4-Mediated Elevation of ETV4 Facilitates Hepatocellular Carcinoma Metastasis by Upregulating PD-L1 and CCL2. J. Hepatol..

[136] Li X., He G., Liu J., Yan M., Shen M., Xu L., An M., Huang J., Gao Z. (2022). CCL2-Mediated Monocytes Regulate Immune Checkpoint Blockade Resistance in Pancreatic Cancer. Int. Immunopharmacol..

[137] Tong J., He M. (2025). Identifying Senescent Cells as Prognostic Biomarkers and Therapeutic Targets of Nasopharyngeal Carcinoma by Integrated Analysis of Single-Cell and Bulk-RNA Sequencing Data. Transl. Cancer Res..

[138] Wang Z., Xie H., Zhou L., Liu Z., Fu H., Zhu Y., Xu L., Xu J. (2016). CCL2/CCR2 Axis Is Associated with Postoperative Survival and Recurrence of Patients with Non-Metastatic Clear-Cell Renal Cell Carcinoma. Oncotarget.

[139] Yang Z., Li H., Wang J., Gao W., Zhao Q., Meng Q., Huang J., Xi Q., Wei J., Yang X. (2025). CCL2/CCR2 Axis Promotes Perineural Invasion of Salivary Adenoid Cystic Carcinoma via ITGβ5-Mediated Nerve-Tumor Interaction. Biochim. Biophys. Acta BBA-Mol. Basis Dis..

[140] Roca H., Varsos Z., Pienta K.J. (2008). CCL2 Protects Prostate Cancer PC3 Cells from Autophagic Death via Phosphatidylinositol 3-Kinase/AKT-Dependent Survivin Up-Regulation. J. Biol. Chem..

[141] Du Z., Sun C., Wu J., Gao H., Wu J., Zhou Y., Wu X., Shen L., Wang Q. (2025). Wogonin Inhibits the Proliferation of Prolactinoma through the PI3K/AKT Signaling Pathway. Front. Pharmacol..

[142] Fang W.B., Jokar I., Zou A., Lambert D., Dendukuri P., Cheng N. (2012). CCL2/CCR2 Chemokine Signaling Coordinates Survival and Motility of Breast Cancer Cells through Smad3 Protein- and P42/44 Mitogen-Activated Protein Kinase (MAPK)-Dependent Mechanisms. J. Biol. Chem..

[143] Kadomoto S., Izumi K., Mizokami A. (2021). Roles of CCL2-CCR2 Axis in the Tumor Microenvironment. Int. J. Mol. Sci..

[144] Prata L.G.P.L., Ovsyannikova I.G., Tchkonia T., Kirkland J.L. (2018). Senescent Cell Clearance by the Immune System: Emerging Therapeutic Opportunities. Semin. Immunol..

[145] Song P., An J., Zou M.-H. (2020). Immune Clearance of Senescent Cells to Combat Ageing and Chronic Diseases. Cells.

[146] Mailloux A.W., Young M.R.I. (2008). Myeloid-derived Suppressor Cells (MDSCs) and Tumor-associated Macrophages (TAMs) Produce CCL22 Which Selectively Recruits Regulatory T-cells (Tregs) to the Tumor Microenvironment. FASEB J..

[147] Yang H., Zhang Q., Xu M., Wang L., Chen X., Feng Y., Li Y., Zhang X., Cui W., Jia X. (2020). CCL2-CCR2 Axis Recruits Tumor Associated Macrophages to Induce Immune Evasion through PD-1 Signaling in Esophageal Carcinogenesis. Mol. Cancer.

[148] De Almeida L.G.N., Thode H., Eslambolchi Y., Chopra S., Young D., Gill S., Devel L., Dufour A. (2022). Matrix Metalloproteinases: From Molecular Mechanisms to Physiology, Pathophysiology, and Pharmacology. Pharmacol. Rev..

[149] Tuomainen A.M., Kormi I., Havulinna A.S., Tervahartiala T., Salomaa V., Sorsa T., Pussinen P.J. (2014). Serum Tissue-Degrading Proteinases and Incident Cardiovascular Disease Events. Eur. J. Prev. Cardiol..

[150] Zhang L., Jiang W., Zhang B., Xu T., Zhou S., Cai M., Chen J., Wu M. (2026). TNFAIP3 Regulates Inflammatory Arthritis through the Differentiation of Monocytes into Macrophages. Asian Pac. J. Allergy Immunol..

[151] He L., Kang Q., Chan K.I., Zhang Y., Zhong Z., Tan W. (2023). The Immunomodulatory Role of Matrix Metalloproteinases in Colitis-Associated Cancer. Front. Immunol..

[152] Mott J.D., Werb Z. (2004). Regulation of Matrix Biology by Matrix Metalloproteinases. Curr. Opin. Cell Biol..

[153] Li S. (2025). Matrix Metalloproteinases as Therapeutic Targets of Neuroinflammation in Alzheimer’s Disease. Biomed. J. Sci. Tech. Res..

[154] Wan J., Zhang G., Li X., Qiu X., Ouyang J., Dai J., Min S. (2021). Matrix Metalloproteinase 3: A Promoting and Destabilizing Factor in the Pathogenesis of Disease and Cell Differentiation. Front. Physiol..

[155] Jiang J., Wu Q., Rajasekaran S., Wu R. (2025). MMP3 at the Crossroads: Linking Molecular Pathways to Disease Diagnosis and Therapy. Pharmacol. Res..

[156] Rivera-Serrano M., Flores-Colón M., Valiyeva F., Meléndez L.M., Vivas-Mejía P.E. (2025). Upregulation of MMP3 Promotes Cisplatin Resistance in Ovarian Cancer. Int. J. Mol. Sci..

[157] Bergers G., Brekken R., McMahon G., Vu T.H., Itoh T., Tamaki K., Tanzawa K., Thorpe P., Itohara S., Werb Z. (2000). Matrix Metalloproteinase-9 Triggers the Angiogenic Switch during Carcinogenesis. Nat. Cell Biol..

[158] O’Reilly M.S., Wiederschain D., Stetler-Stevenson W.G., Folkman J., Moses M.A. (1999). Regulation of Angiostatin Production by Matrix Metalloproteinase-2 in a Model of Concomitant Resistance. J. Biol. Chem..

[159] Suhaimi S.A., Chan S.C., Rosli R. (2020). Matrix Metallopeptidase 3 Polymorphisms: Emerging Genetic Markers in Human Breast Cancer Metastasis. J. Breast Cancer.

[160] De Oliveira R.F., Salazar M., Matos L., Almeida H., Rodrigues A.R., Gouveia A.M. (2024). High Copper Levels Induce Premature Senescence in 3T3-L1 Preadipocytes. Biochim. Biophys. Acta BBA-Mol. Cell Res..

[161] Martini H., Birch J., Marques F.D.M., Victorelli S., Lagnado A.B., Pirius N., Franco A.C., Lee G., Han Y., Rowsey J.L. (2026). Mitochondrial Metabolism and Epigenetic Crosstalk Drive SASP. Nature.

[162] Di Micco R., Krizhanovsky V., Baker D., d’Adda Di Fagagna F. (2021). Cellular Senescence in Ageing: From Mechanisms to Therapeutic Opportunities. Nat. Rev. Mol. Cell Biol..

[163] Dou X., Fu Q., Long Q., Liu S., Zou Y., Fu D., Xu Q., Jiang Z., Ren X., Zhang G. (2023). PDK4-Dependent Hypercatabolism and Lactate Production of Senescent Cells Promotes Cancer Malignancy. Nat. Metab..

[164] Li L., Dong J., Xu C., Wang S. (2025). Lactate Drives Senescence-Resistant Lineages in Hepatocellular Carcinoma via Histone H2B Lactylation of NDRG1. Cancer Lett..

[165] Zuo R., Wu C., Lin Z., Kong L., Wang F., Zhang X., Wei J., Lin Y., Yang E., Kang Q. (2026). Investigating the Mechanistic Link Between Lactate-Induced Histone Lactylation and Cellular Senescence in Osteoarthritis Chondrocytes: Implications for Therapy. Int. J. Biol. Sci..

[166] Sun C., Li J., Dong L., Mou Y., Zhang B., Song X. (2025). Lactylation: A Novel Epigenetic Regulator of Cellular Senescence. Aging Dis..

[167] Wiley C.D., Sharma R., Davis S.S., Lopez-Dominguez J.A., Mitchell K.P., Wiley S., Alimirah F., Kim D.E., Payne T., Rosko A. (2021). Oxylipin Biosynthesis Reinforces Cellular Senescence and Allows Detection of Senolysis. Cell Metab..

[168] Kim J., Vaish V., Feng M., Field K., Chatzistamou I., Shim M. (2016). Transgenic Expression of Cyclooxygenase-2 (COX2) Causes Premature Aging Phenotypes in Mice. Aging.

[169] Dagouassat M., Gagliolo J.-M., Chrusciel S., Bourin M.-C., Duprez C., Caramelle P., Boyer L., Hue S., Stern J.-B., Validire P. (2013). The Cyclooxygenase-2–Prostaglandin E2 Pathway Maintains Senescence of Chronic Obstructive Pulmonary Disease Fibroblasts. Am. J. Respir. Crit. Care Med..

[170] Gonçalves S., Yin K., Ito Y., Chan A., Olan I., Gough S., Cassidy L., Serrao E., Smith S., Young A. (2021). COX2 Regulates Senescence Secretome Composition and Senescence Surveillance through PGE2. Cell Rep..

[171] Tan Y.J., Conley T.E., Yao F., García-Marqués F.J., Akinyemi D.E., Dinh V.V., Wang Q., Bermudez A., Kim J., Belk J.A. (2026). Restored Clearance of Senescent Neutrophils by Tissue-Resident Macrophages Limits Organ Aging. Science.

[172] St. Sauver J.L., Weston S.A., Atkison E.J., Mc Gree M.E., Mielke M.M., White T.A., Heeren A.A., Olson J.E., Rocca W.A., Palmer A.K. (2021). Biomarkers of cellular senescence and risk of death in humans. Aging Cell.

[173] Zhao S., Kuo C.-L., Lenze E.J., Wetherell J.L., Haynes L., El-Ahmad P., Fortinsky R., Kuchel G., Harris T., Diniz B.S. (2026). A Deep-Learning Based Biomarker of Systemic Cellular Senescence Burden to Predict Mortality and Health Outcomes. Aging Cell.

[174] Justice J.N., Nambiar A.M., Tchkonia T., LeBrasseur N.K., Pascual R., Hashmi S.K., Prata L., Masternak M.M., Kritchevsky S.B., Musi N. (2019). Senolytics in Idiopathic Pulmonary Fibrosis: Results from a First-in-Human, Open-Label, Pilot Study. EBioMedicine.

[175] ClinicalTrials.gov Targeting Pro-Inflammatory Cells in Idiopathic Pulmonary Fibrosis: A Human Trial (IPF). Identifier NCT02874989. NCT02874989.

[176] Nambiar A., Kellogg D., Justice J., Goros M., Gelfond J., Pascual R., Hashmi S., Masternak M., Prata L., LeBrasseur N. (2023). Senolytics Dasatinib and Quercetin in Idiopathic Pulmonary Fibrosis: Results of a Phase I, Single-Blind, Single-Center, Randomized, Placebo-Controlled Pilot Trial on Feasibility and Tolerability. EBioMedicine.

[177] Zwicker J.I., Schlechter B.L., Stopa J.D., Liebman H.A., Aggarwal A., Puligandla M., Caughey T., Bauer K.A., Kuemmerle N., Wong E. (2019). Targeting Protein Disulfide Isomerase with the Flavonoid Isoquercetin to Improve Hypercoagulability in Advanced Cancer. JCI Insight.

[178] ClinicalTrials.gov Cancer Associated Thrombosis and Isoquercetin (CATIQ). Identifier NCT02195232. NCT02195232.

[179] ClinicalTrials.gov Dasatinib and Quercetin with CAR-T Therapy for the Treatment of Patients with Relapsed or Refractory Multiple Myeloma. Identifier NCT06940297. NCT06940297.

[180] Huntington S.F., Schuster S.J., Ding W., Koehler A.B., Brander D.M., Rosenthal A.C., Leis J.F., Tun H.W., Moustafa M.A., Iqbal M. (2023). DTRMWXHS-12, a Novel Bruton Tyrosine Kinase Inhibitor, in Combination with Everolimus and Pomalidomide in Patients with Relapsed/Refractory Lymphomas: An Open-label, Multicenter, Phase 1a/1b Study. Am. J. Hematol..

[181] ClinicalTrials.gov Safety Study of BTK Inhibitor, DTRMWXHS-12, Used Singly or in Combination, in CLL and B-Cell Lymphomas. Identifier NCT02900716. NCT02900716.

[182] Prat A., Brase J.C., Cheng Y., Nuciforo P., Paré L., Pascual T., Martínez D., Galván P., Vidal M., Adamo B. (2019). Everolimus plus Exemestane for Hormone Receptor-Positive Advanced Breast Cancer: A PAM50 Intrinsic Subtype Analysis of BOLERO-2. Oncologist.

[183] Yardley D.A., Noguchi S., Pritchard K.I., Burris H.A., Baselga J., Gnant M., Hortobagyi G.N., Campone M., Pistilli B., Piccart M. (2013). Everolimus Plus Exemestane in Postmenopausal Patients with HR+ Breast Cancer: BOLERO-2 Final Progression-Free Survival Analysis. Adv. Ther..

[184] Chinnaiyan P., Won M., Wen P.Y., Rojiani A.M., Werner-Wasik M., Shih H.A., Ashby L.S., Michael Yu H.-H., Stieber V.W., Malone S.C. (2018). A Randomized Phase II Study of Everolimus in Combination with Chemoradiation in Newly Diagnosed Glioblastoma: Results of NRG Oncology RTOG 0913. Neuro-Oncology.

[185] ClinicalTrials.gov Everolimus, Temozolomide, and Radiation Therapy in Treating Patients with Newly Diagnosed Glioblastoma Multiforme (RTOG 0913). Identifier NCT01062399. NCT01062399.

[186] ClinicalTrials.gov EVERolimus and LenvAtinib Versus Everolimus for Bone Sarcoma. Identifier NCT07619950. NCT07619950.

[187] ClinicalTrials.gov A Study of Setanaxib Co-Administered with Pembrolizumab in Patients with Recurrent or Metastatic Squamous Cell Carcinoma of Head and Neck (SCCHN). Identifier NCT05323656. NCT05323656.

[188] Sylvester A.L., Zhang D.X., Ran S., Zinkevich N.S. (2022). Inhibiting NADPH Oxidases to Target Vascular and Other Pathologies: An Update on Recent Experimental and Clinical Studies. Biomolecules.

[189] ClinicalTrials.gov A Trial of Setanaxib in Patients with Primary Biliary Cholangitis (PBC) and Liver Stiffness (TRANSFORM). Identifier NCT05014672. NCT05014672.

[190] Xia M., Sui Z. (2009). Recent Developments in CCR2 Antagonists. Expert Opin. Ther. Pat..

[191] ClinicalTrials.gov Neoadjuvant Nivolumab with CCR2/5-Inhibitor or Anti-IL-8) for Non-Small Cell Lung Cancer (NSCLC) or Hepatocellular Carcinoma (HCC). Identifier NCT04123379. NCT04123379.

